# Elevated Expression and Activity of Sodium Leak Channel Contributes to Neuronal Sensitization of Inflammatory Pain in Rats

**DOI:** 10.3389/fnmol.2021.723395

**Published:** 2021-08-27

**Authors:** Jia Li, Yali Chen, Jin Liu, Donghang Zhang, Peng Liang, Peilin Lu, Jiefei Shen, Changhong Miao, Yunxia Zuo, Cheng Zhou

**Affiliations:** ^1^Department of Anesthesiology, West China Hospital, Sichuan University, Chengdu, China; ^2^Laboratory of Anaesthesia and Critical Care Medicine, Translational Neuroscience Center, West China Hospital, Sichuan University, Chengdu, China; ^3^Department of Anesthesiology, Xi’an Jiaotong University-Affiliated Honghui Hospital, Xi’an, China; ^4^Laboratory of Oral Diseases & National Clinical Research Center for Oral Diseases and Department of Prosthodontics, West China Stomatology Hospital of Sichuan University, Chengdu, China; ^5^Department of Anesthesiology, Zhongshan Hospital, Fudan University, Shanghai, China

**Keywords:** NALCN, electrophysiology, inflammatory pain, neuronal sensitization, substance P

## Abstract

Inflammatory pain encompasses many clinical symptoms, and there is no satisfactory therapeutic target. Neuronal hyperexcitability and/or sensitization of the primary nociceptive neurons in the dorsal root ganglion (DRG) and spinal dorsal horn are critical to the development and maintenance of inflammatory pain. The sodium leak channel (NALCN), a non-selective cation channel, mediates the background Na^+^ leak conductance and controls neuronal excitability. It is unknown whether abnormal activity of NALCN mediates the pathological process of inflammatory pain. Complete Freund’s adjuvant (CFA) was injected into the left footpad of rats to induce inflammatory pain. The thresholds of mechanical and thermal sensation and spontaneous pain behaviors were assessed. The expression of NALCN in DRG and spinal dorsal cord was measured. NALCN currents and the contribution of NALCN to neuronal excitability in the DRG and spinal dorsal cord were recorded using whole-cell patch-clamping recording. NALCN was abundantly expressed in neurons of the DRG and spinal dorsal cord. In acutely isolated DRG neurons and spinal cord slices from rats with CFA-induced inflammatory pain, NALCN currents and neuronal excitability were increased. Subsequently, intrathecal and sciatic nerve injection of NALCN-small interfering RNA (siRNA) decreased NALCN mRNA and reverted NALCN currents to normal levels, and then reduced CFA-induced neuronal excitability and alleviated pain symptoms. Furthermore, pain-related symptoms were significantly prevented by the NALCN-shRNA-mediated NALCN knockdown in DRG and spinal cord. Therefore, increased expression and activity of NALCN contributed to neuronal sensitization in CFA-induced inflammatory pain. NALCN may be a novel molecular target for the control of inflammatory pain.

## Introduction

Inflammatory pain is induced by neural injury or disease that affects approximately 20% of the general population; however, a large proportion of patients with inflammatory pain is treated ineffectively (Goldberg and McGee, 2011). Several causes may contribute to the development of inflammatory pain. Current therapeutics for inflammatory pain mainly include non-steroidal anti-inflammatory drugs and opioids, but the effects are not satisfactory and their use are also limited because of side effects (Weinblatt, 1991; Mehta, 2007; Carter et al., 2014). Therefore, the development of novel drugs is clinically important for the treatment of inflammatory pain.

Neuronal hyperexcitability and/or sensitization of the primary nociceptive neurons in the dorsal root ganglion (DRG) and spinal dorsal horn is critical to the development and maintenance of inflammatory pain (Herrmann et al., 2017; Amsalem et al., 2018). However, the key molecular target that regulates the intrinsic excitability of neurons in DRG and spinal dorsal horn during the development of inflammatory pain has yet to be identified.

The sodium leak channel (NALCN), a non-selective background leak cation channel, is widely expressed in the nervous system, including the DRG and spinal cord (Lu et al., 2007; Zhang et al., 2021). NALCN can regulate the neuronal resting membrane potential (RMP; Lu et al., 2007; Cochet-Bissuel et al., 2014), which is associated with neuronal excitability and rhythm in the central nervous system (Lu et al., 2007). Abnormal function of NALCN induces many physiological dysfunctions, such as respiratory failure in mice (Yeh et al., 2017), disrupted circadian rhythm in *Drosophila melanogaster* (Flourakis et al., 2015), motor dysfunction in *Caenorhabditis elegans* (Gao et al., 2015), and cognitive defects in humans (Bend et al., 2016; Lozic et al., 2016). For sensory functions, NALCN can regulate the excitability of spinal cord projection neurons (Ford et al., 2018). Overexpression of NALCN in nociceptors of African highveld mole-rat-induced depolarization of the neuronal RMP (Eigenbrod et al., 2019). However, it is unclear whether the expression and the function of NALCN in the DRG and spinal cord contribute to the initiation and maintenance of inflammatory pain.

Substance P (SP), a neuropeptide and neurotransmitter, is synthesized in cell bodies of pain-sensing fibers of DRG, and then it is transported and released in the spinal dorsal horn (Hökfelt et al., 1975). SP exerts its effects via neurokinin 1 (NK1) receptors, which is involved in the transmission of nociceptive sensations (Andoh et al., 1996; Man et al., 2012). SP-NK1 receptor signaling also modulates the activity of many ion channels, including Na_v_1.8 (Cang et al., 2009), transient receptor potential cation channel subfamily V member 1 (TRPV1) (Zhang et al., 2007; Lapointe et al., 2015), and potassium channels (Sculptoreanu et al., 2009). Interestingly, NALCN is activated by SP in the neurons of both hippocampus and ventral tegmental area in mice (Lu et al., 2007, 2009; Ou et al., 2020). SP enhances respiration by activating NALCN in the pre-Bötzinger complex and the retrotrapezoid nucleus (Ren, 2011; Yeh et al., 2017). However, it is still not known whether NALCN in DRG and spinal cord neurons is the molecular target of SP in inflammatory pain.

In the present study, we hypothesized that the NALCN is a potent regulator of neuronal activity in the DRG and spinal dorsal cord in a rat model of complete Freund’s adjuvant (CFA)-induced inflammatory pain. Our findings suggest that NALCN might be an important molecular target for the control of pain induced by inflammation.

## Materials and Methods

### Animals

All the study protocols were approved by the Animal Ethics Committee of West China Hospital of Sichuan University (Chengdu, Sichuan, China) (Approval No. 2018181A). The protocols were conducted in accordance with the Animal Research: Reporting of *in vivo* Experiments (ARRIVE) guidelines. Neonatal (postnatal days 7–10, PD7∼10, male and female) Sprague-Dawley rats and adult (∼6 weeks, male) rats were used for electrophysiological recordings. Adult (6–8 weeks, male) rats were used for behavioral tests. All rats were housed under controlled conditions (temperature 22–24°C; humidity, 45–55%; and a 12/12-h light–dark cycle) with free access to food and water. Neonatal rats were kept with their mothers. Every effort was made to minimize the number of rats used and to minimize their pain.

### Chemicals

Substance P (MedChemExpress LLC, Monmouth Junction, NJ, United States) was dissolved in saline and intrathecally injected at a concentration of 1 μg/μl. The NK1 receptor antagonist L-703606 (Sigma-Aldrich, St. Louis, MO, United States) was dissolved in saline and intrathecally injected at a concentration of 6 μg/μl.

### The CFA-Induced Inflammatory Pain Model in Rats

Under 2% isoflurane anesthesia, the rats were subcutaneously injected with CFA (Sigma-Aldrich, St. Louis, MO, United States, 1 mg ml^–1^) in the left footpad, using a 28-gauge needle. The injected volumes were 100 and 10 μl for adult and neonatal rats, respectively.

### Injection of Interventions

Small interfering RNAs (siRNA), modified by 3′-AlexaFluor488 (QIAGEN, Germantown, MD, United States), were dissolved in RNase-free water (NALCN-siRNA: 5′-UUGGCGUUGGUAUCUUCGCTT-3′; control-siRNA: catalog No. 1027419, QIAGEN, United States). *In vivo* SilenceMag^TM^ transfection reagent (OZ Biosciences, Marseille, France) was mixed with NALCN-siRNA or control-siRNA to a concentration of 1 μg/μl. Then, NALCN-siRNA or control-siRNA was injected into the sciatic nerve (1 μl for neonatal and 2 μl for adult rats) and subarachnoid space between L5 and L6 (5 μl for neonatal and 10 μl for adults). Briefly, rats were anesthetized with 2% isoflurane. Intraneural injection was at mid-part of sciatic nerve, using a glass micropipette connected to a Hamilton syringe. Then a magnet (SilenceMag^TM^ kit) was placed at the body surface that corresponded to the targeted site for at least 1 h after injection. For intrathecal injection, the rats were restrained in the horizontal position, and a 25-gauge needle connected to a Hamilton syringe was slowly inserted through punctured skin into the subarachnoid space between L5 and L6. A sudden flick of the tail indicates a successful puncture into the subarachnoid space. The needle was kept in place for at least 60 s after the injection to avoid an outflow.

Dorsal root ganglion microinjection was performed as described previously (Zhao et al., 2013). Laminectomy was performed to expose the DRG (left L4-L5), and 2 μl pAAV2-H1-shRNA-(NALCN)-CAG-eGFP or pAAV2-scrambled-CAG-eGFP virus (2 × 10^13^ TU/ml) (Taitool Bioscience, Shanghai, China) was unilaterally injected into each DRG at a constant speed of 0.2 μl/min with a glass micropipette connected to a Hamilton syringe affixed by a stereotaxic syringe holder (SYS-Micro4, WPI, Sarasota, FL, United States). The injection needle was carefully removed after 10 min. The experiments were performed in all rats 4 weeks after the injection. The intrathecal injection method of AAV was the same with that of siRNA.

### Behavioral Tests

Von Frey filaments and the Hargreaves test (heat) were used to measure mechanical and thermal hyperalgesia. For von Frey test, rats were placed individually in a Plexiglas chamber on an elevated metal mesh floor and habituated for 30 min. The plantar surface of the hind limb was stimulated with a series of von Frey filaments (0.07, 0.16, 0.4, 0.6, 1, 1.4, 2, 4, 6, 8, 10, and 15 g). The absolute withdrawal threshold was determined using the up-down method (Dixon, 1991). In Hargreaves test, rats were habituated in glass chambers on an elevated transparent glass platform. A beam of radiant heat from a thermal stimulator was focused on the plantar surface of the hind limb. The latency from the onset of radiant heat to an aversive response was defined as the thermal withdrawal latency. The cut-off time of radiant heat was set at 20 s to prevent tissue damage. The withdrawal threshold in the von Frey test and the thermal withdrawal latency in the Hargreaves test were measured three times daily, and the averages were calculated (with at least 10-min intervals between measurements). The behavioral tests were performed before the injection of CFA (baseline) and at 2 h, 8 h, and day 1 to day 12 daily after the injection of CFA. The experimenter was blinded to the groups of rats.

For the evaluation of CFA and SP-induced spontaneous aversive behaviors, the rats were firstly acclimated in the transparent recording chamber for 30 min. After intrathecal injection of SP or intraplantar injection of CFA, nociceptive behaviors were recorded, including paw licking, lifting, and shaking, and the duration of nociceptive behaviors (Rivat et al., 2018). The experimenter was blinded to the groups of rats.

### Preparation of Spinal Cord Slices for Patch-Clamp Recording

Neonatal rats (PD7∼10) were anesthetized by inhalation of 2% isoflurane. The lumbar enlargement of the spinal cord was quickly excised, fixed in agarose gel (2%), and mounted on the chamber of a vibratome (VT1000A; Leica, Wetzlar, Germany). The chamber was filled with chilled (0–4°C) cutting solution containing (in mM): 260 sucrose, 3 KCl, 5 MgCl_2_, 1 CaCl_2_, 1.25 NaH_2_PO_4_, 26 NaHCO_3_, 10 glucose, and 1 kynurenic acid (pH 7.4). Sagittal 300-μm thick spinal cord slices were cut and incubated at 36–37°C for 1 h, and then stored at room temperature (24–26°C) with incubation solution containing (in mM): 130 NaCl, 3 KCl, 2 MgCl_2_, 2 CaCl_2_, 1.25 NaH_2_PO_4_, 26 NaHCO_3_, and 10 glucose (pH 7.4). The incubation solution was equilibrated with 95% O_2_/5% CO_2_. After incubation, spinal cord slices were placed in the recording chamber at room temperature.

### Acutely Isolated DRG Neurons

Acutely isolated DRG neurons were prepared as previously reported (Xu and Huang, 2002). Briefly, the rats were anesthetized with 2% isoflurane. L4-L6 DRGs were removed and digested sequentially in 2% papain for 30 min and 2% collagenase I for 30 min at 37°C. Isolated neurons were then plated on glass coverslips coated with poly-D-lysine and laminin, and incubated with neuronal culture medium (Neurobasal medium, 2% B27, 1% Glutamax), aerated with 95% O_2_/5% CO_2_. Patch-clamp recordings were performed ∼2 h later.

### Patch-Clamp Recordings

Spinal cord slices or acutely isolated DRG neurons with coverslips were mounted in a recording chamber and submerged in continuously perfused incubation solution (∼2 ml/min), bubbled with 95% O_2_/5% CO_2_. The incubation solution contained (in mM): 130 NaCl, 3 KCl, 2 MgCl_2_, 2 CaCl_2_, 1.25 NaH_2_PO_4_, 26 NaHCO_3_, and 10 glucose (pH 7.4). L4-L6 DRG and spinal dorsal horn (lamina I-II) neurons were visualized and identified by the shape using infrared differential interference contrast microscopy. Electrophysiological recordings were conducted using an Axopatch 700B amplifier and a Digidata 1440 digitizer linked to a computer running pClamp 10.2 software (Molecular Devices, Sunnyvale, CA, United States). Recordings were sampled at 20 kHz and filtered at 10 kHz. All recordings were performed in the whole-cell configuration. The resistance of patch electrodes was 4–6 MΩ. Current-clamp recordings were performed to record action potentials using an internal solution containing (in mM): 120 KCH_3_SO_3_, 4 NaCl, 1 MgCl_2_hin CaCl_2_, 10 HEPES, 10 EGTA, 3 Mg-ATP, and 0.3 GTP-Tris, pH 7.3 (adjusted with KOH). For the recordings in the current-clamp mode, the rheobase was determined by applying a series of short depolarizing current steps (from −10 to 300 pA for DRG, from −5 to 200 pA for spinal dorsal cord) until a single action potential was generated. The duration of depolarizing current was 800 ms for each current step, and the interval was 5 pA for spinal dorsal cord and 10 pA for DRG. Voltage-clamp recordings were established to measure NMDG- (140 mM), Gd^3+^- (50 μM), and SP-sensitive (10 μM) holding currents using an internal solution containing (in mM): 104 CsCH_3_SO_3_, 1 MgCl_2_, 0.5 CaCl_2_, 30 TEA-Cl, 10 EGTA, 3 Mg-ATP, 0.3 GTP-Tris, 10 HEPES, pH 7.3 (adjusted with CsOH). The series resistance was compensated by 70–75%, and data were rejected if the series resistance exceeded 20 MΩ. To block K^+^ channels, synaptic GABAergic and AMPA inputs, 25-mM TEA, 5-mM 4-Aminopyridine (4-AP), 10-μM CNQX, and 10-μM bicuculline were added to the incubation solution. For NALCN current recording, 500-nM tetrodotoxin (TTX) was added to the incubation solution as previously described (Yang et al., 2020).

### Real-Time Polymerase Chain Reaction

Total RNA was extracted using an Eastep^®^ Super RNA extraction kit (Promega, Shanghai, China), and reverse transcription of total RNA was performed with a GoScript^TM^ Reverse Transcription Kit (Promega, Shanghai, China). Real-time polymerase chain reaction (PCR) was performed using GoTaq^®^ qPCR Master Mix (Promega, Shanghai, China) and specific primers (Sangon Biotech, Shanghai, China) according to the instructions of the manufacturer. PCR cycling parameters were set as follows: 40 cycles at 95°C for 30 s, 60°C for 30 s, and then 72°C for 30 s. The Ct values of test genes were normalized to the value for GAPDH. The primers used to detect NALCN, SP, and GAPDH mRNA were:

NALCN forward (5′-GTCCTGACGAATCTCTGTCAGA-3′);

NALCN reverse (5′- CTGAGATGACGCTGATGATGG-3′);

SP forward (5′-GACAGTGACCAAATCAAGGAGG -3′);

SP reverse (5′-GAGGAATCAGCATCCCGTTTG-3′);

GAPDH forward (5′-GACATGCCGCCTGGAGAAAC-3′); and

GAPDH reverse (5′-AGCCCAGGATGCCCTTTAGT-3′).

### Western Blotting

The lumbar enlargement of the spinal cord and L4-L6 DRGs were rapidly homogenized and lysed on ice. The protein concentration was determined with a BCA protein assay kit (Beyotime, Shanghai, China). The protein was electrophoresed on a 7.5% sodium dodecyl sulfate polyacrylamide gel and then transferred to polyvinyl difluoride (PVDF) membranes (Bio-Rad, Hercules, CA, United States). The PVDF membranes were incubated overnight at 4°C with primary antibodies against NALCN (1:1,000, Alomone Labs, Jerusalem, Israel) and β-actin (1:5,000, Abcam, Cambridge, United Kingdom). Then, membranes were incubated with the secondary antibody (1:4,000, Cell Signaling Technology, Beverly, MA, United States) at ambient temperature for 2 h. The protein bands were quantified using ImageJ software (National Institutes of Health, Bethesda, MD, United States).

### Immunofluorescence Staining

The rats were deeply anesthetized by inhalation of 2% isoflurane and transcardially perfused with phosphate-buffered saline (PBS, pH 7.4) for 5 min, and then followed by 4% paraformaldehyde until muscle twitching stopped and liver tissue was hardened (5 min) as previously described (Chalermpalanupap et al., 2018; Kumar et al., 2019). L4–L6 DRGs and the lumbar enlargement of the spinal cord were removed and soaked in 4% formalin overnight at 4°C and then in 30% sucrose until the tissue sank to the bottom. The spinal cords and DRGs were cut into 12-μm-thick sequential sections using a freezing microtome (CM1850; Leica, Buffalo Grove, IL, United States). Sections were double labeled by incubating them overnight at 4°C with primary antibodies against NALCN (1:400, Alomone Labs, Jerusalem, Israel), TRPV1 (1:800, Abcam, Cambridge, United Kingdom), SP (1:200, Abcam, Cambridge, United Kingdom), neurofilament 200 (NF200, 1:800, Millipore, Burlington, MA, United States), isolectin B4 (IB4)-FITC-conjugated 488 (1:400, Sigma, St. Louis, MO, United States), NeuN (1:400, Sigma, St. Louis, MO, United States), and vesicular glutamate transporter 2 (VGLUT2, 1:200, Novus, Centennial, CO, United States). Fluorescent secondary antibodies (1:400, Jackson Immuno Research, West Grove, PA, United States) were added to 5% bovine serum albumin and incubated for 2 h at room temperature. For cell-counting analysis, five rats were included in each group. Twenty sections of L4–L6 DRGs of each rat were randomly selected for statistical analysis. The percent of co-expression of NALCN and SP, TRPV1, NF200, or IB4-positive cells was obtained. All images were captured using a Zeiss Axio Imager Z.2.

### Statistical Analysis

All figures were prepared using GraphPad Prism 8.0 software (GraphPad Software, San Diego, CA, United States). Statistical tests were carried out using SPSS 22.0 software (IBM, Armonk, NY, United States). A Shapiro–Wilk normality test was used to assess the normality of the data distribution. Normally distributed data were presented as the mean ± standard deviation (SD) and were analyzed with student’s *t*-test. Non-normally distributed data were presented as median (quartile), and the Mann–Whitney *U* test was used. The behavioral experiment data were analyzed by two-way repeated measures ANOVA; if significance was established, *post hoc* Holm–Sidak multiple comparisons were performed. The real-time PCR data were analyzed by paired, unpaired student’s *t*-tests or two-way ANOVA; if significance was established, *post hoc* Holm–Sidak multiple comparisons were performed. The Western blotting data were analyzed by two-way (compared between groups) ANOVA; if significance was established, *post hoc* Holm–Sidak multiple comparisons were performed. The electrophysiology data were analyzed by paired, unpaired Student’s *t*-tests, or two-way ANOVA; if significance was established, *post hoc* Holm–Sidak multiple comparisons were performed. All statistical methods used for individual analyses were indicated in the figure legends. The level of significance was set as *p* < 0.05.

## Results

### Relative Expression of NALCN and SP Increased After the Injection of CFA in Adult Rats

**FIGURE 1:**
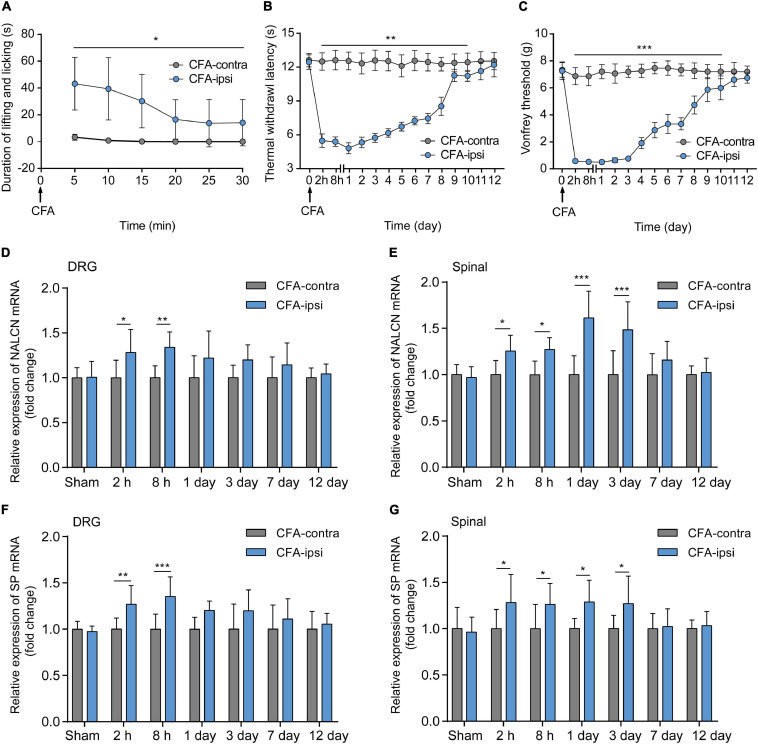
Relative expression of sodium leak channel (NALCN) and substance P (SP) increase after injection of CFA. **(A)** Time course of spontaneous pain behavior (lifting/licking/flinching behaviors), following CFA injection (*n* = 10, by two-way repeated measures ANOVA, Holm–Sidak test). **(B)** Time course of thermal hyperalgesia following CFA injection (*n* = 10, by two-way repeated measures ANOVA, Holm–Sidak test). **(C)** Time course of mechanical allodynia, following CFA injection (*n* = 10, by two-way repeated measures ANOVA, Holm–Sidak test). **(D)** Changes in NALCN mRNA level in DRG (*n* = 10, by two-way ANOVA, Holm–Sidak test). **(E)** Changes in NALCN mRNA in spinal dorsal horn (*n* = 10, by two-way ANOVA, Holm–Sidak test). **(F)** Changes in SP mRNA in DRG (*n* = 10, by two-way ANOVA, Holm–Sidak test). **(G)** Changes in SP mRNA expression in spinal dorsal horn (*n* = 10, by two-way ANOVA, Holm–Sidak test). CFA-contra: CFA-contralateral side; CFA-ipsi: CFA-ipsilateral side; DRG: dorsal root ganglion; SP: substance P. Data are presented as mean ± SD. *p < 0.05, **p < 0.01, ***p < 0.001.

Spontaneous aversive behaviors were observed for 30 min after the injection of CFA, including lifting and licking of the injected foot (Figure 1A, *n* = 10, *p* < 0.05). Compared with the CFA-contralateral side, thermal hyperalgesia and mechanical allodynia were detected in the CFA-ipsilateral side from 2 h to day 10 after the CFA injection (Figures 1B,C, *n* = 10, *p* < 0.05, respectively). The most pronounced mechanical allodynia and/or thermal hyperalgesia was detected on day 1 after CFA injection. The relative mRNA level of NALCN (Figure 1D) and SP (Figure 1F) in the DRG from the CFA-ipsilateral side was increased at 2 h (*n* = 10, *p* = 0.011 for NALCN; *p* = 0.008 for SP) and 8 h (*n* = 10, *p* = 0.001 for NALCN; *p* < 0.001 for SP) after CFA injection, compared to the CFA-contralateral side. In the spinal dorsal horn, NALCN (Figure 1E) and SP (Figure 1G) mRNA levels were increased at 2 h (*n* = 6, *P* = 0.026 for NALCN; *P* = 0.021 for SP), 8 h (*n* = 10, *P* = 0.015 for NALCN; *P* = 0.037 for SP), day 1 (*n* = 10, *p* < 0.001 for NALCN; *P* = 0.016 for SP) and day 3 (*n* = 10, *p* < 0.001 for NALCN; *P* = 0.029 for SP) after CFA injection.

**FIGURE 2:**
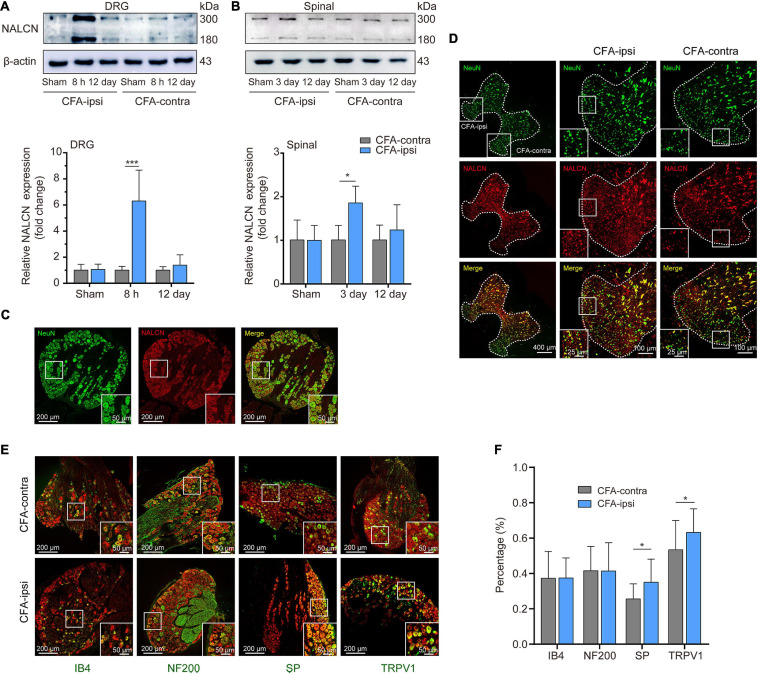
Expression of NALCN increases in DRG and spinal dorsal horn after CFA injection in rats. **(A)** Representative immunoblotting image and statistical analysis indicated that the level of NALCN protein in DRG was upregulated at 8 h after CFA injection (*n* = 5, by two-way ANOVA, Holm–Sidak test). **(B)** The level of NALCN protein in spinal cord was upregulated at day 3 after CFA injection (*n* = 5, by two-way ANOVA, Holm–Sidak test). **(C)** Double immunofluorescence staining performed with an NALCN antibody in DRG at 8 h after CFA injection. NALCN (red) and neuronal marker NeuN (green) immunoreactivity showed NALCN mostly colocalized with NeuN. **(D)** Double immunofluorescence staining of spinal cord at day 3 after CFA injection showed NALCN immunoreactivity mostly colocalized with NeuN. **(E)** Double immunostaining shows colocalization of NALCN (red) with IB4 (green), NF200 (green), SP (green), and TRPV1 (green) in DRG neurons. **(F)** Percent of neuronal markers of the total population of DRG neurons. Five animals were used to obtain counts (unpaired *t*-test). IB4, isolectin B4; NF200, neurofilament 200 kDa; SP, substance P; TRPV1, transient receptor potential vanilloid 1. Data are presented as mean ± SD. *p < 0.05, ***p < 0.001.

The NALCN non-selective protein was also evaluated at the highest mRNA expression time point. Expression of NALCN protein was 6.3 ± 2.4-fold greater in the CFA-ipsilateral side than that in the CFA-contralateral side in the DRG at 8 h after CFA injection (Figure 2A, *n* = 5, *p* < 0.001) and 1.8 ± 0.4-fold greater in the spinal dorsal cord at day 3 after CFA injection (Figure 2B, *n* = 5, *P* = 0.01). By fluorescence staining, NALCN was widely expressed in nearly all neurons of the DRG (Figure 2C) and spinal dorsal cord (Figure 2D). NALCN was widely co-expressed with SP, NF200, IB4, and TRPV1 in the DRG (Figure 2E). The percent of DRG neurons that co-expressed SP and NALCN (25.7% ± 8.5% vs. 35% ± 13.1%, *n* = 11–16 sections, *P* = 0.032) and co-expressed TRPV1 and NALCN (53.4% ± 16.6% vs. 63.2% ± 13.3%, *n* = 30–29 sections, *P* = 0.015) were increased on the CFA-ipsilateral side (Figure 2F). In the spinal dorsal cord, NALCN was widely expressed in the SP-positive area (Supplementary Figure 1). Notably, the specificity of the NALCN primary antibody was confirmed by both Western blotting and fluorescence staining (Supplementary Figure 2).

### Na^+^-Mediated Currents in DRG Neurons Functionally Increase After CFA Injection

**FIGURE 3:**
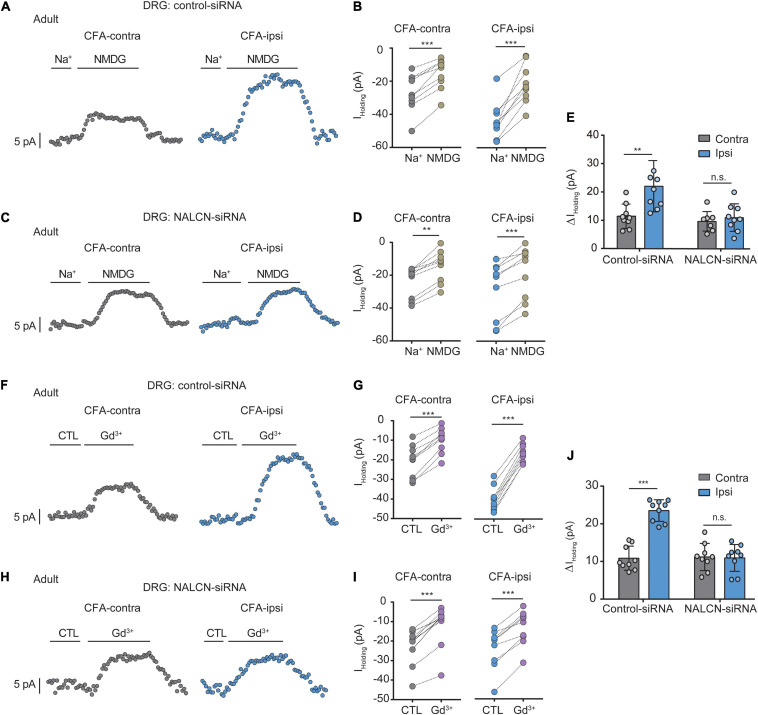
Na^+^-mediated currents (*I*_holding_) and gadolinium (Gd^3+^)-inhibited *I*_holding_ increase in small and medium neurons of DRG after injection of CFA in adult rats. Currents were recorded at holding potential of –60 mV. **(A)** Representative real-time analysis of *I*_holding_ in small and medium DRG neurons from the rats that received control-siRNA combined with injection of CFA. *I*_holding_ was extracted by replacing extracellular Na^+^ with NMDG. **(B)** The *I*_holding_ in small and medium DRG neurons of control siRNA-treated rats were analyzed before and after replacement with NMDG. **(C)** Representative real-time analysis of holding currents in small and medium DRG neurons from the rats that received NALCN-siRNA combined with injection of CFA. *I*_holding_ was extracted by replacing extracellular Na^+^ with NMDG. **(D)** The *I*_holding_ in small and medium DRG neurons of NALCN siRNA-treated rats were analyzed before and after replacement with NMDG. **(E)** The *I*_holding_ increased more in the DRG neurons of CFA-ipsi side than those of CFA-contra side from the control-siRNA-treated rats; treatment of NALCN-siRNA diminished the difference in *I*_holding_ between CFA-ipsi and CFA-contra sides (*n* = 9, by two-way ANOVA, Holm–Sidak test). **(F)** Representative real-time analysis of Gd^3+^-inhibited *I*_holding_ in small and medium DRG neurons from the rats that received control-siRNA combined with injection of CFA. **(G)** The *I*_holding_ in small and medium DRG neurons of control siRNA-treated rats was analyzed before and after perfusion of Gd^3+^. **(H)** Representative real-time analysis of Gd^3+^-inhibited *I*_holding_ in small and medium DRG neurons from the rats that received NALCN-siRNA combined with injection of CFA. **(I)** The *I*_holding_ in small and medium DRG neurons of NALCN siRNA-treated rats was analyzed before and after perfusion of Gd^3+^. **(J)** Gd^3+^-inhibited *I*_holding_ increased more in the DRG neurons of CFA-ipsi side than those of CFA-contra side from the control-siRNA-treated rats; treatment of NALCN-siRNA diminished the difference in Gd^3+^-inhibited *I*_holding_ between CFA-ipsi and CFA-contra sides (*n* = 9, by two-way ANOVA, Holm–Sidak test). CFA-contra: CFA-contralateral side; CFA-ipsi: CFA-ipsilateral side; CTL: control, before perfusion of Gd^3+^; DRG: dorsal root ganglion. Data are present as mean ± SD. **p < 0.01, ***p < 0.001, n.s.: no significance.

To determine the function of NALCN in CFA-induced pain, we intrathecally and intraneurally injected NALCN-siRNA and/or control-siRNA in adult rats at day 3 after CFA injection. The Na^+^-mediated current (*I*_holding_) was recorded at a holding potential of −60 mV by replacing extracellular Na^+^ with NMDG (Figures 3A,C). For the adult rats that received control-siRNA, the total change in *I*_holding_ was larger in small- and medium-sized DRG neurons from the CFA-ipsilateral side compared with the CFA-contralateral side (Figures 3B,E, left, 21.8 ± 9. vs. 11.2 ± 4.3 pA, *n* = 9, *P* = 0.001). Treatment of NALCN-siRNA diminished the difference in *I*_holding_ between the CFA-ipsilateral and CFA-contralateral sides in small- and medium-sized DRG neurons (Figures 3D,E, right, 10.9 ± 4.9 vs. 9.6 ± 3.4 pA, *n* = 9, *P* = 0.865). Gadolinium (Gd^3+^) is a non-selective blocker of NALCN. The *I*_holding_ was blocked by Gd^3+^ (Figures 3F,H). The total change of Gd^3+^-mediated inhibition of *I*_holding_ was greater in small- and medium-sized DRG neurons of the CFA-ipsilateral side compared with the CFA-contralateral side in rats that received control-siRNA (Figures 3G,J, left, 23.1 ± 2.9 vs. 10.6 ± 3.2 pA, *n* = 9, *p* < 0.001). Treatment of NALCN-siRNA diminished the difference in Gd^3+^-inhibited *I*_holding_ between the CFA-ipsilateral and CFA-contralateral sides in small- and medium-sized DRG neurons (Figures 3I,J, right, 10.6 ± 3.5 vs. 10.9 ± 3.6 pA, *n* = 9, *P* = 0.984). No difference was found between Gd^3+^-mediated current inhibition and that produced by siRNA (Supplementary Figure 3A, 12.45 ± 2.27 vs. 10.89 ± 5.31 pA, *n* = 9, *P* = 0.431).

**FIGURE 4:**
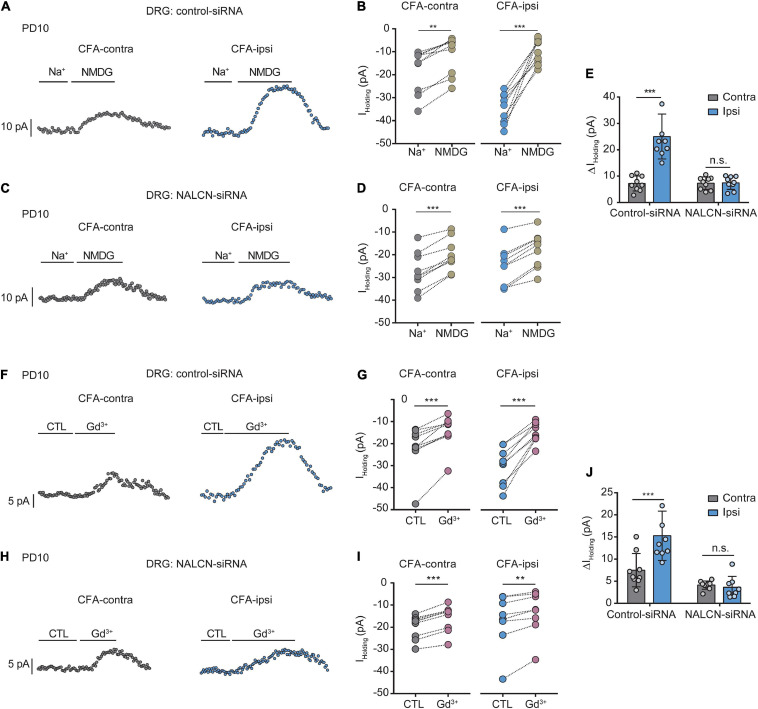
Na^+^-mediated currents and gadolinium (Gd^3+^)-inhibited holding currents in DRG neurons increase after injection of CFA in neonatal rats. NALCN-siRNA and/or control-siRNA were intrathecally and intraneurally injected into the subarachnoid space and sciatic nerve in P7 rats; CFA was injected 2 days later. **(A)** Representative real-time analysis of *I*_holding_ in small and medium neurons from the rats that received control-siRNA combined with injection of CFA. The *I*_holding_ was recorded at holding potential of –60 mV and extracted by replacing extracellular Na^+^ with NMDG. **(B)** The *I*_holding_ in small and medium DRG neurons of control siRNA-treated rats was analyzed before and after replacement with NMDG. **(C)** Representative real-time analysis of *I*_holding_ in small and medium neurons from the rats that received NALCN-siRNA combined with an injection of CFA. **(D)** The *I*_holding_ in small and medium DRG neurons of NALCN siRNA-treated rats was analyzed before and after replacement with NMDG. **(E)** The *I*_holding_ increased more in the DRG neurons of CFA-ipsi side than those of CFA-contra side from the control-siRNA-treated rats; treatment of NALCN-siRNA diminished the difference of *I*_holding_ between CFA-ipsi and CFA-contra side (*n* = 9, by two-way ANOVA, Holm–Sidak test). **(F)** Representative real-time analysis of Gd^3+^-inhibited *I*_holding_ in small and medium DRG neurons from the rats that received control-siRNA combined with injection of CFA. **(G)** The *I*_holding_ in small and medium DRG neurons of control siRNA-treated rats was analyzed before and after perfusion of Gd^3+^. **(H)** Representative real-time analysis of Gd^3+^-inhibited *I*_holding_ in small and medium DRG neurons from the rats that received NALCN-siRNA combined with injection of CFA. **(I)** The *I*_holding_ in small and medium DRG neurons of NALCN siRNA-treated rats was analyzed before and after perfusion of Gd^3+^. **(J)** Gd^3+^-inhibited *I*_holding_ was increased in DRG neurons of CFA-ipsi side than those of CFA-contra side from control siRNA-treated rats, but no difference was shown between CFA-ipsi side and CFA-contra sides from the NALCN-siRNA-treated rats (*n* = 9, by two-way ANOVA, Holm–Sidak test). CFA-contra: CFA-contralateral side; CFA-ipsi: CFA-ipsilateral side; CTL: control, before perfusion of Gd^3+^; DRG: dorsal root ganglion; RMP: rest membrane potential; AP: action potential. Data are presented as mean ± SD. **p < 0.01, ***p < 0.001, n.s.: no significance.

Similar experiments were also performed in neonatal rats (PD7∼10). We intrathecally and intraneurally injected NALCN-siRNA and/or control-siRNA in neonatal rats at day 3 after CFA injection. Change of NALCN mRNA was confirmed in neonatal rats after CFA injection. The level of NALCN mRNA was increased at 8 h in the DRG after injection of CFA in neonatal rats (Supplementary Figure 4A, left, *n* = 9, *P* = 0.040). The level of NALCN mRNA was decreased on day 3 in the DRG after injection of NALCN-siRNA (Supplementary Figure 4B, left, *n* = 12, *p* < 0.001). Whole-cell patch-clamp recordings were performed on acutely isolated small and medium neurons of DRG (8 h after CFA injection). The *I*_holding_ was recorded at a holding potential of −60 mV by replacing normal extracellular Na^+^ with NMDG (Figures 4A,C). For the rats that received control-siRNA, the total change in *I*_holding_ was larger in the neurons of DRG from the CFA-ipsilateral side, compared with the CFA-contralateral side (Figures 4B, left; Figures 4E, left, 24.6 ± 8.5 vs. 7. ± 2.8 pA, *n* = 9, *p* < 0.001). Treatment of NALCN-siRNA diminished the difference of *I*_holding_ between the CFA-ipsilateral and CFA-contralateral sides in DRG neurons (Figure 4D, right; Figure 4E, right, 7.2 ± 2.5 vs. 7.1 ± 2.4 pA, *n* = 9, *P* = 0.997). The *I*_holding_ was blocked by Gd^3+^ (Figures 4F,H). The total change of Gd^3+^-mediated inhibition of *I*_holding_ was greater in small- and medium-sized DRG neurons of the CFA-ipsilateral side, compared with the CFA-contralateral side in rats that received control-siRNA (Figures 4G,J, left, 15.2 ± 5.6 vs. 7.4 ± 3.8 pA, *n* = 9, *p* < 0.001). Treatment with NALCN-siRNA diminished the difference in Gd^3+^-inhibited *I*_holding_ between the two sides in small- and medium-sized DRG neurons (Figures 4I,J, right, 3.5 ± 2.5 vs. 4. ± 0.9 pA, *n* = 9–8, *P* = 0.952). No difference was found between Gd^3+^-mediated current inhibition and that produced by siRNA [Supplementary Figure 3B, 9.96 (9.54, 12.51) vs. 13.42 (8.82, 14.64) pA, *n* = 8, *P* = 0.382].

### Elevated Activity of NALCN Contributes to Excitability of DRG Neurons

**FIGURE 5:**
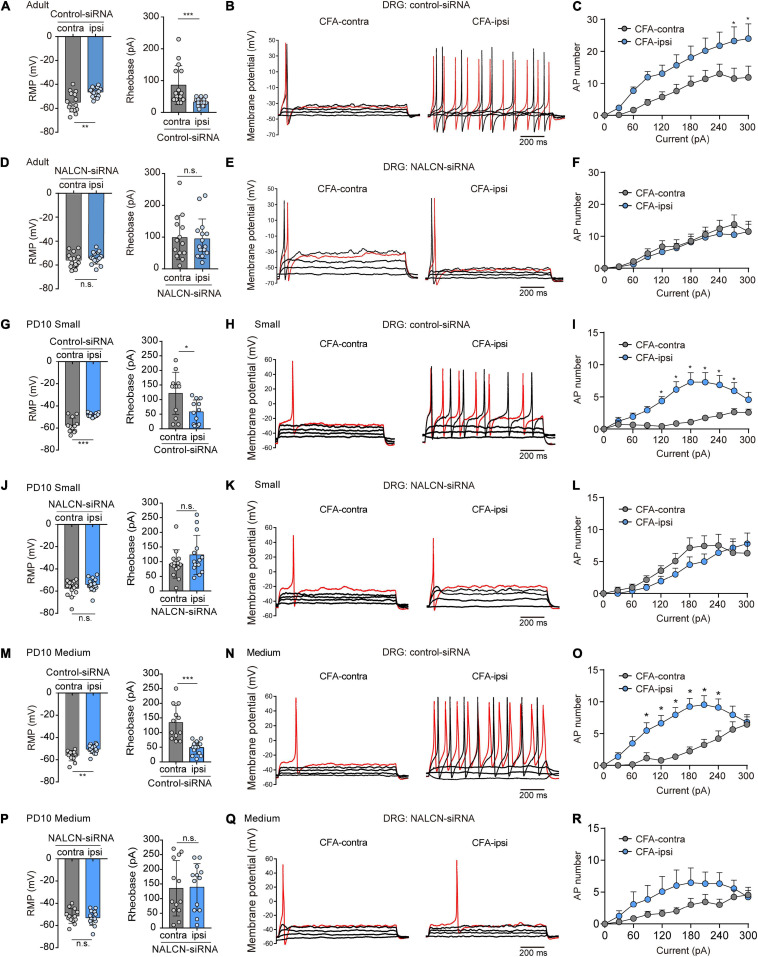
Sodium leak channel enhances the excitability of small- and medium-sized DRG neurons after CFA injection in adult and neonatal rats. **(A,D)** Excitability of small and medium neurons in DRG compared between CFA-contralateral and CFA-ipsilateral sides based on RMP and rheobase from the rats that received control-siRNA and/or NALCN-siRNA combined with injection of CFA in adult rats (*n* = 14–15, unpaired *t*-test). **(B,E)** Representative traces of action potentials in the DRG neurons in control-siRNA and/or NALCN siRNA-treated rats. Red traces were the action potentials when receiving the same current injection. **(C,F)** Numbers of APs were increased more in small and medium DRG neurons of CFA-ipsi side than those of CFA-contra side from control siRNA-treated rats [**(C)**, *n* = 14, by two-way ANOVA, Holm–Sidak test], but no difference was shown between CFA-ipsi side and CFA-contra side from the NALCN-siRNA-treated rats [**(F)**, *n* = 15, by two-way ANOVA, Holm–Sidak test], respectively. **(G,J)** Excitability of small neurons in DRG compared between CFA-contralateral and CFA-ipsilateral sides based on RMP and rheobase from the rats that received control-siRNA and/or NALCN-siRNA combined with injection of CFA (*n* = 12, unpaired *t*-test). **(H,K)** Representative traces of action potentials in the small neurons of DRG from the rats that received control-siRNA and/or NALCN-siRNA combined with injection of CFA. Red trace was the action potential when receiving the same current injection. **(I,L)** Numbers of APs were increased more in small DRG neurons of CFA-ipsi side than those of CFA-contra side from control siRNA-treated rats [**(I)**, *n* = 12, by two-way ANOVA, Holm–Sidak test], but no difference was shown between CFA-ipsi side and CFA-contra side from the NALCN-siRNA-treated rats [**(L)**, *n* = 12, by two-way ANOVA, Holm–Sidak test], respectively. **(M,P)** Excitability of medium neurons in DRG compared between CFA-contralateral and CFA-ipsilateral sides based on RMP and rheobase from the rats that received control-siRNA and/or NALCN-siRNA combined with injection of CFA (*n* = 12–14, unpaired *t*-test). **(N,Q)** Action potential traces of medium neurons of DRG from the rats that received control-siRNA and/or NALCN-siRNA combined with injection of CFA. Red trace was the action potential when receiving the same current injection. **(O,R)** Numbers of APs increased more in medium DRG neurons of CFA-ipsi side than those of CFA-contra side from control siRNA-treated rats [**(O)**, *n* = 12, by two-way ANOVA, Holm–Sidak test], but no difference was shown between CFA-ipsi side and CFA-contra side from the NALCN-siRNA-treated rats [**(R)**, *n* = 14, by two-way ANOVA, Holm–Sidak test], respectively. CFA-contra: CFA-contralateral side; CFA-ipsi: CFA-ipsilateral side; DRG: dorsal root ganglion; RMP: rest membrane potential; AP: action potential. Data are presented as mean ± SD. *p < 0.05, ***p < 0.001, n.s.: no significance.

NALCN-siRNA was used to test whether NALCN contributes to the excitability of DRG neurons after CFA injection. For adult rats, representative traces of action potentials of DRG neurons were shown in Figures 5B,E. With treatment of control-siRNA, small- and medium-sized neurons in the DRG from the CFA-ipsilateral side were hyperactive, as evidenced by depolarized RMP (Figure 5A, left, −46.4 ± 3.9 vs. −54.9 ± 8.1 mV, *n* = 15, *P* = 0.001), decreased rheobase (Figure 5D, left, 32.7 ± 12.2 vs. 85.3 ± 61.3 pA, *n* = 15, *p* < 0.001), and increased the number of AP spikes (Figure 5C). With treatment of NALCN-siRNA, the RMP (Figure 5A, right, −55.4 ± 6.1 vs. −53.7 ± 5.1 mV, *n* = 14–15, *P* = 0.417), rheobase (Figure 5D, right, 98.6 ± 69.9 vs. 94.7 ± 61.9 pA, *n* = 14–15, *P* = 0.940), and the number of AP spikes (Figure 5F) did not differ between the CFA-ipsilateral and CFA-contralateral sides for small- and medium-sized DRG neurons.

For neonatal rats, representative traces of action potentials of small, medium, large DRG neurons were shown in Figures 5H,K,N,Q. With treatment of control-siRNA, the small neurons in DRG from the CFA-ipsilateral side were hyperactive, as evidenced by depolarized RMPs (Figure 5G, left, −48.3 ± 1.9 vs. −57.5 ± 6.5 mV, *n* = 12, *p* < 0.001), decreased rheobase (Figure 5G, right, 58.3 ± 40.5 vs. 121.7 ± 72 pA, *n* = 12, *P* = 0.015) and increased the number of AP spikes (Figure 5I). The medium-sized neurons in DRG from the CFA-ipsilateral side were also hyperactive (RMP in Figure 5M, left, −50.4 ± 4. vs. −56.4 ± 4.6 mV, *n* = 12–14, *P* = 0.002; rheobase in Figure 5M, right, 48.6 ± 22.1 vs. 134.2 ± 59.2 pA, *n* = 12–14, *p* < 0.001), with the increased number of AP spikes (Figure 5O). However, for large neurons, the RMP (Supplementary Figure 5A, left, *n* = 13, *P* = 0.870), rheobase (Supplementary Figure 5A, right, *n* = 13, *P* = 0.773) and the number of AP spikes (Supplementary Figure 5C) did not significantly differ between the CFA-ipsilateral and CFA-contralateral sides. With treatment of NALCN-siRNA, the RMP, rheobase, and the number of AP spikes did not differ between the two sides for small (Figure 5J, *n* = 14, *P* = 0.184; *P* = 0.171, respectively, Figure 5L), medium (Figure 5P, *n* = 13, *P* = 0.538; *P* = 0.912, respectively, Figure 5R) or large neurons (Supplementary Figure 5D, *n* = 13, *P* = 0.482; *P* = 0.771, respectively, Supplementary Figure 5F). These results indicated that the increased excitability of small- and medium-sized neurons in the DRG of the CFA-ipsilateral side, at least partly, resulted from the increased NALCN activity.

### Na^+^-Mediated Currents in Spinal Dorsal Horn Are Increased After CFA Injection

**FIGURE 6:**
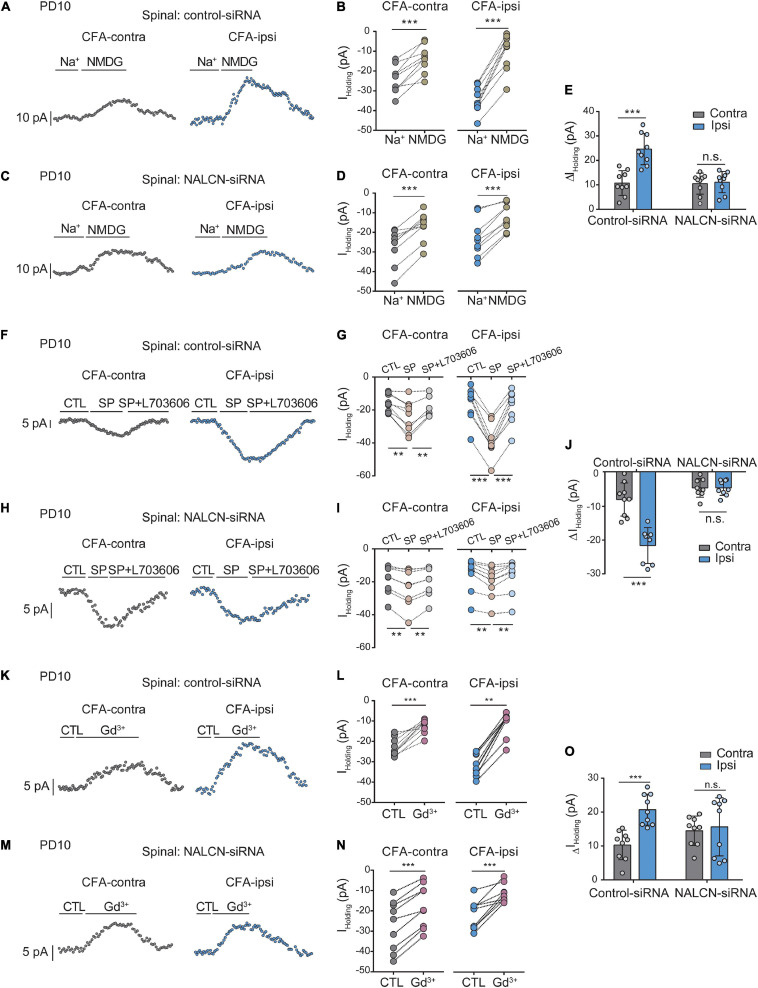
Na^+^-mediated currents and gadolinium (Gd^3+^)-inhibited *I*_holding_ in neurons of spinal cord increase after injection of CFA. Sensory neurons of spinal dorsal cord were recorded at holding potential of –60 mV. **(A)** The representative real-time analysis of *I*_holding_ in neurons from the rats that received control-siRNA combined with injection of CFA. The *I*_holding_ was extracted by replacing extracellular Na^+^ with NMDG. **(B)** The *I*_holding_ in spinal cord neurons of control siRNA-treated rats was analyzed before and after replacement with NMDG. **(C)** The representative real-time analysis of holding currents in neurons from the rats that received NALCN-siRNA combined with injection of CFA. The *I*_holding_ was extracted by replacing extracellular Na^+^ with NMDG. **(D)** The *I*_holding_ in spinal cord neurons of NALCN siRNA-treated rats was analyzed before and after replacement with NMDG. **(E)** The *I*_holding_ increased more in the spinal cord neurons of CFA-ipsi side than those of CFA-contra side from the control-siRNA-treated rats; treatment of NALCN-siRNA diminished the difference in *I*_holding_ between CFA-ipsi and CFA-contra sides (*n* = 9, by two-way ANOVA, Holm–Sidak test). **(F)** The representative real-time analysis of *I*_holding_ in neurons from the rats that received control-siRNA combined with injection of CFA. SP-evoked *I*_holding_ was recorded at holding potential of –60 mV. L703606 (10 μM) blocked SP-evoked *I*_holding_. **(G)** The *I*_holding_ in spinal dorsal horn neurons of control siRNA-treated rats was analyzed before and after perfusion of SP and L703606. **(H)** The representative real-time analysis of *I*_holding_ in neurons from the rats that received NALCN-siRNA combined with injection of CFA. SP-evoked *I*_holding_ was recorded at holding potential of –60 mV. L703606 (10 μM) blocked SP-evoked *I*_holding_. **(I)** The *I*_holding_ in spinal dorsal horn neurons of NALCN siRNA-treated rats was analyzed before and after perfusion of SP and L703606. **(J)** SP-evoked *I*_holding_ increased more in the spinal cord neurons of CFA-ipsi side than those of CFA-contra side from the control-siRNA-treated rats; treatment of NALCN-siRNA diminished the difference in SP-evoked *I*_holding_ between CFA-ipsi and CFA-contra side (*n* = 8–9, by two-way ANOVA, Holm–Sidak test). **(K)** Representative real-time analysis of Gd^3+^-inhibited *I*_holding_ in neurons of dorsal spinal cord from the rats that received control-siRNA combined with injection of CFA. **(L)** The *I*_holding_ in neurons of dorsal spinal cord of control siRNA-treated rats was analyzed before and after perfusion of Gd^3+^. **(M)** Representative real-time analysis of Gd^3+^-inhibited *I*_holding_ in neurons of dorsal spinal cord from the rats that received NALCN-siRNA combined with injection of CFA. **(N)** The *I*_holding_ in neurons of dorsal spinal cord of NALCN siRNA-treated rats was analyzed before and after perfusion of Gd^3+^. **(O)** Gd^3+^-inhibited *I*_holding_ increased more in neurons of dorsal spinal cord of CFA-ipsi side than those of CFA-contra side from control siRNA-treated rats, but no difference was shown between CFA-ipsi side and CFA-contra side from the NALCN-siRNA-treated rats (*n* = 9, by two-way ANOVA, Holm–Sidak test). CFA-contra: CFA-contralateral side; CFA-ipsi: CFA-ipsilateral side; DRG: dorsal root ganglion; CTL: control, before perfusion of SP or Gd^3+^; RMP: rest membrane potential. Data are present as mean ± SD. **p < 0.01, ***p < 0.001, n.s.: no significance.

The level of NALCN mRNA was increased on day 1 after injection of CFA in the spinal dorsal cord of neonatal rats (Supplementary Figure 4A, right, *n* = 9, *P* = 0.003). Injection of NALCN-siRNA led to a decreased level of NALCN mRNA on day 3 in the spinal dorsal cord of neonatal rats (Supplementary Figure 4B, right, *n* = 11, *p* < 0.001). Whole-cell patch-clamp recordings were performed on acutely isolated spinal cord slices on day 1 after injection of CFA. The *I*_holding_ was recorded at a holding potential of −60 mV and measured by replacing normal extracellular Na^+^ with NMDG (Figures 6A,C). With treatment of control-siRNA, the change of *I*_holding_ was larger in the neurons from spinal dorsal cord (Figures 6B,E, left, 24. ± 6.3 vs. 10.4 ± 5 pA, *n* = 9, *p* < 0.001) of the CFA-ipsilateral side, compared with the CFA-contralateral side. Treatment of NALCN-siRNA diminished the difference in *I*_holding_ between the two sides in spinal dorsal horn neurons (Figures 6D,E, right, 10.8 ± 4.2 vs.10.2 ± 4.2 pA, *n* = 9, *P* = 0.957).

The *I*_holding_ was enhanced by perfusion of SP onto neurons of the spinal dorsal horn and was blocked by the NK1 receptor antagonist L703606 (Figures 6F,H). After control-siRNA treatment, the SP-evoked *I*_holding_ was larger in spinal dorsal horn neurons of the CFA-ipsilateral side (Figures 6G,J, left, −22.1 ± 5.4 vs. −8.2 ± 5 pA, *n* = 9–8, *p* < 0.001). Treatment with NALCN-siRNA diminished the difference in SP-evoked *I*_holding_ of neurons of the spinal dorsal horn between the CFA-ipsilateral and CFA-contralateral sides (Figures 6I,J, right, −4.7 ± 2.8 vs. −4.7 ± 2.8 pA, *n* = 8–9, *p* > 0.999). These results indicated that the NALCN-mediated current was increased after CFA injection, which may strengthen the response to SP in the spinal dorsal horn neurons but can be reduced by NALCN-siRNA.

Next, the *I*_holding_ was blocked with Gd^3+^in spinal cord neurons (Figures 6K,M). The Gd^3+^-mediated inhibition of *I*_holding_ was greater in spinal dorsal cord neurons on the CFA-ipsilateral side of rats that treated with control-siRNA (Figures 6L,O, left, 20.8 ± 4.6 vs. 10.2 ± 4.4 pA, *n* = 9, *p* < 0.001). For the rats that treated with NALCN-siRNA, no difference was found in Gd^3+^-inhibited *I*_holding_ of spinal dorsal cord neurons between the CFA-ipsilateral and the CFA-contralateral sides (Figures 6N,O, right, 9.6 ± 2.9 vs. 10.4 ± 5.8 pA, *n* = 9, *P* = 0.919). No difference was found between Gd^3+^-mediated current inhibition and that produced by siRNA (Supplementary Figure 3C, 10.36 ± 2.04 vs. 12.63 ± 2.38 pA, *n* = 8–9, *P* = 0.052).

### Elevated Activity of NALCN Contributes to Excitability of Sensory Neurons in the Spinal Dorsal Horn

**FIGURE 7:**
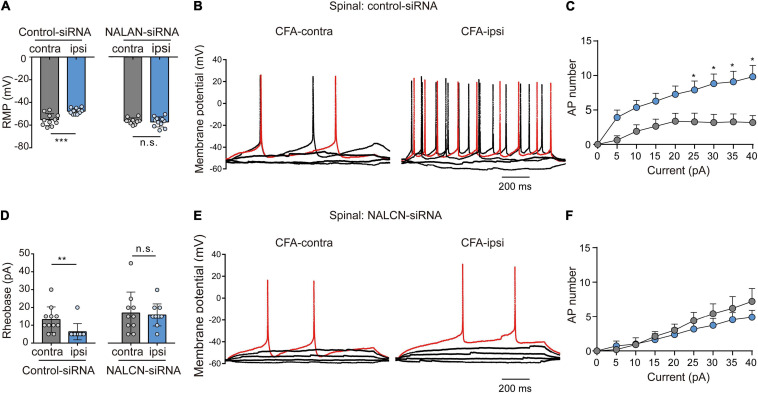
Sodium leak channel enhances the excitability of sensory neurons of spinal dorsal horn after injection of CFA. **(A,D)** Excitability of spinal neurons compared between CFA-contralateral and CFA-ipsilateral sides based on RMP and rheobase from the rats that received control-siRNA and/or NALCN-siRNA combined with injection of CFA (*n* = 11, unpaired *t*-test; *n* = 11, Mann–Whitney *U* test, unpaired *t*-test, respectively). **(B,E)** Representative traces of action potentials in the neurons of spinal dorsal horn from the rats that received control-siRNA and/or NALCN-siRNA combined with injection of CFA. Red trace was the action potentials when receiving the same current injection. **(C,F)** Numbers of APs increased more in spinal dorsal horn neurons of CFA-ipsi side than those of CFA-contra side from control siRNA-treated rats [**(C)**, *n* = 12, by two-way ANOVA, Holm–Sidak test], but no difference was shown between CFA-ipsi side and CFA-contra sides from the NALCN-siRNA-treated rats [**(F)**, *n* = 14, by two-way ANOVA, Holm–Sidak test, respectively]. CFA-contra: CFA-contralateral side; CFA-ipsi: CFA-ipsilateral side; RMP: rest membrane potential. Data are present as mean ± SD. *p < 0.05, **p < 0.01, ***p < 0.001, n.s.: no significance.

Representative traces of action potentials of spinal dorsal horn neurons were shown in Figures 7B,E. The neurons from the CFA-ipsilateral side were hyperactive in rats treated with control-siRNA (depolarized RMP, Figure 7A, left, −47.7 ± 2.6 vs. −55.2 ± 5.3 mV, *n* = 11, *p* < 0.001; decreased rheobase, Figure 7D, left, 5 (5,5) vs. 10 (10,15) pA, *n* = 11, *P* = 0.002; the increased number of AP spikes, Figure 7C). However, the RMP (Figure 7A, right, −57.3 ± 4.5 vs. −56.5 ± 2.8 mV, *n* = 10–11, *P* = 0.654), rheobase (Figure 7D, right, 15.9 ± 6.3 vs. 17. ± 11.8 pA, *n* = 10–11, *P* = 0.792), and the number of AP spikes (Figure 7F) did not differ between the CFA-ipsilateral and CFA-contralateral sides in NALCN-siRNA-treated rats. Thus, the increased excitability of neurons in the spinal dorsal horn of the CFA-ipsilateral side, at least partly, resulted from the increased NALCN activity.

### Knockdown Expression of NALCN in the DRG and Spinal Cord Reduces SP- and CFA-Induced Pain Behaviors

**FIGURE 8:**
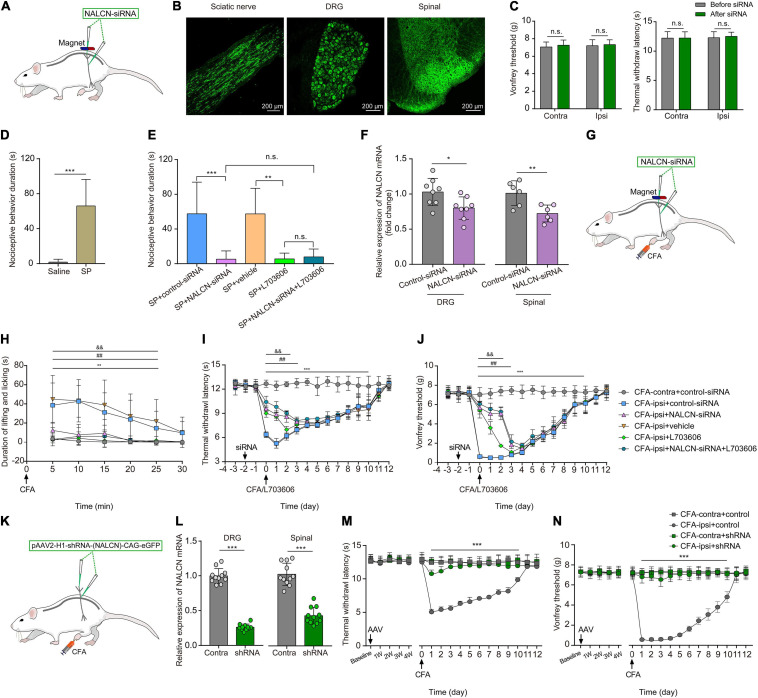
The knockdown of expression of NALCN in DRG and spinal cord alleviates pain behaviors induced by SP or CFA. **(A)** siRNA administration methods used in adult rats. NALCN-siRNA and/or control-siRNA were intrathecally and intraneurally injected into the subarachnoid space and sciatic nerve in adult rats. **(B)** Alexa Fluor 488-positive fluorescence was found in sciatic nerve, DRG, and spinal dorsal cord after injection of NALCN-siRNA. **(C)** NALCN-siRNA did not affect the thermal hyperalgesia and mechanical allodynia of normal rats. **(D)** Intrathecal injection of SP induced pain-related behaviors in rats, including biting, licking, and scratching at the abdomen and hind portions of the body (*n* = 10, unpaired *t*-test). **(E)** The duration of biting, licking, and scratching within the first 5 min after injection of SP decreased in the rats that received NALCN-siRNA, L703606, siRNA + L703606 (*n* = 10, by two-way ANOVA, Holm–Sidak test). **(F)** The level of NALCN mRNA was decreased at day 3 after injection of NALCN-siRNA in DRG and spinal cord compared with control-siRNA (*n* = 8–6, unpaired *t*-test). **(G)** Modeling and siRNA administration methods used in adult rats. NALCN-siRNA and/or control-siRNA were intrathecally and intraneurally injected into the subarachnoid space and sciatic nerve in adult rats. **(H)** NALCN-siRNA, L703606, and siRNA + L703606 alleviated spontaneous pain behavior (lifting/licking/flinching behaviors) within the first 25 min, following CFA injection (*n* = 10, two-way repeated measures ANOVA, Holm–Sidak test). **(I)** NALCN-siRNA, L703606, and siRNA + L703606 alleviated thermal hyperalgesia at day 0–3 after CFA injection (*n* = 10, two-way repeated measures ANOVA, Holm–Sidak test). **(J)** NALCN-siRNA, L703606, and siRNA + L703606 alleviated mechanical allodynia at day 0–3 after CFA injection (*n* = 10, two-way repeated measures ANOVA, Holm–Sidak test). **(K)** Representative cartoons of pAAV2-H1-shRNA-(NALCN)-CAG-eGFP injection. **(L)** NALCN mRNA was decreased in DRG of the AAV-NALCN-shRNA group as compared with AAV-scrambled-shRNA group (left panel, *n* = 11, unpaired *t*-test). NALCN mRNA was decreased in dorsal spinal cord of the AAV-NALCN-shRNA group as compared with the AAV-scrambled-shRNA group (right panel, *n* = 11, unpaired *t*-test). **(M,N)** Normal sensory function in thermal test [**(M)**, left panel, *n* = 10] or von Frey [**(N)**, left panel, *n* = 10] was unchanged during the 4 weeks after AAV-NALCN-shRNA injection, compared with AAV-scrambled-shRNA or baseline. NALCN-shRNA alleviated thermal hyperalgesia [**(M)**, right panel, *n* = 10, CFA-ipsi + shRNA vs. CFA-ipsi + control, two-way repeated measures ANOVA, Holm–Sidak test] and mechanical allodynia [**(N)**, right panel, *n* = 10, CFA-ipsi + shRNA vs. CFA-ipsi + control, two-way repeated measures ANOVA, Holm–Sidak test] throughout after CFA, compared with scrambled shRNA. DRG: dorsal root ganglion; SP: substance P; CFA-contra: CFA-contralateral side; CFA-ipsi: CFA-ipsilateral side; ANOVA: analysis of variance. Data are presented as mean ± SD. *p < 0.05, **p < 0.01, ***p < 0.001 CFA-contra + control-siRNA vs. CFA-ipsi + control-siRNA; ^##^*p* < 0.01 CFA-ipsi + control-siRNA vs. CFA-ipsi + NALCN-siRNA; ^& &^*p* < 0.01 CFA-ipsi + vehicle vs. CFA-ipsi + L703606.

To investigate whether NALCN was involved in SP- and CFA-induced pain behaviors *in vivo*, NALCN-siRNA and/or control-siRNA was injected intrathecally and intraneurally into the subarachnoid space and sciatic nerve of adult rats (Figure 8A). Alexa Fluor 488-positive fluorescence was detected in the sciatic nerve, DRG, and spinal dorsal cord on day 2 after siRNA injection (Figure 8B). NALCN-siRNA-treated rats did not display any difference in the mechanical sensation threshold [Figure 8C, *n* = 10, before NALCN-siRNA vs. after NALCN-siRNA (*P* = 0.707 for contralateral side; *P* = 0.856 for ipsilateral side)] or thermal sensation threshold [Figure 8C, *n* = 10, before NALCN-siRNA vs. after NALCN-siRNA (*P* = 0.999 for contralateral side; *P* = 0.833 for ipsilateral side)] on day 3 after injection of NALCN-siRNA. SP-induced acute aversive behaviors in normal rats (Figure 8D, *n* = 10, *p* < 0.001), whereas the knockdown of NALCN expression in the DRG and spinal cord reduced the duration that the rats spent biting, licking, and scratching within the first 5 min after intrathecal injection of SP (Figure 8E, *n* = 10, *p* < 0.001). Intrathecal injection of the NK1 receptor antagonist L703606 also reduced the duration of aversive behaviors after SP injection (Figure 8E, *n* = 10, *P* = 0.001), but L703606 did not further decrease the duration of aversive responses in rats that had received NALCN-siRNA (Figure 8E, *p* > 0.999). Accordingly, expression of NALCN mRNA was decreased with 20.97% ± 15.95% in the DRG (Figure 8F, left, *n* = 8, *P* = 0.022) and with 27.46% ± 11.99% in the spinal dorsal horn (Figure 8F, right, *n* = 6, *P* = 0.008) on day 3 after injection of NALCN-siRNA.

To determine whether NALCN was involved in CFA-induced pain *via* the NK1 receptor, NALCN-siRNA and/or control-siRNA was injected into the subarachnoid space and sciatic nerve of adult rats. Two days later, 100 μLCFA was injected into the left hind foot (Figure 8G). Spontaneous pain within the first 25 min (Figure 8H, *n* = 10, *p* < 0.05), thermal hyperalgesia (Figure 8I, *n* = 10, *p* < 0.05) and mechanical allodynia (Figure 8J, *n* = 10, *p* < 0.05) of the injected hind foot were alleviated by preinjection of NALCN-siRNA compared with preinjection of control-siRNA-treated rats. The therapeutic effects of NALCN-siRNA lasted until day 3 after the CFA injection. Preinjection of the NK1 receptor antagonist L703606 also reduced spontaneous pain (Figure 8H, *n* = 10, *p* < 0.05), thermal hyperalgesia (Figure 8I, *n* = 10, *p* < 0.05), and mechanical allodynia (Figure 8J, *n* = 10, *p* < 0.05) until day 2 in the rats compared with preinjection of vehicle rats. However, L703606 did not further alleviate spontaneous pain (Figure 8H, *n* = 10, *p* > 0.05), thermal hyperalgesia (Figure 8I, *n* = 10, *p* > 0.05) or mechanical allodynia (Figure 8J, *n* = 10, *p* > 0.05) in rats that had received NALCN-siRNA.

The degradation of NALCN-siRNA is fast, and the action time of NALCN-siRNA is short after injection. To test the role of NALCN in maintenance of CFA-induced pain, pAAV2-H1-shRNA-(NALCN)-CAG-eGFP (AAV-NALCN-shRNA) or pAAV2-scrambled-CAG-eGFP (AAV-scrambled-shRNA) virus was injected into DRG and subarachnoid space of adult rats (Figure 8K). The NALCN mRNA level was also significantly decreased both in the DRG (Figure 8L, left, *n* = 11, *p* < 0.001) and the dorsal spinal cord (Figure 8L, right, *n* = 11, *p* < 0.001) by AAV-NALCN-shRNA at 4 weeks after injection. Accordingly, CFA-induced thermal hyperalgesia (Figure 8M, *n* = 10, *p* < 0.05) and mechanical allodynia (Figure 8N, *n* = 10, *p* < 0.05) were prevented throughout by the AAV-NALCN-shRNA but not the AAV-scrambled-shRNA.

## Discussion

The present study suggests that increased expression and function of NALCN in the DRG and spinal dorsal horn were associated with the behavioral hyperalgesia induced by inflammatory pain. The knockdown of NALCN expression relieved CFA-induced pain behaviors and suppressed the excitability of sensory neurons in the DRG and spinal cord. Our findings indicate that NALCN regulates the excitability of sensory neurons and thus may cause inflammatory pain. Therefore, NALCN is suggested to be a potential molecular target for the control of inflammatory pain. Considering the important functions of NALCN in neuronal rhythms, such as respiration, peripherally restricted NALCN blockers might be a novel potential therapy for inflammatory pain.

Despite the fundamental roles of the DRG and spinal dorsal horn neurons in inflammatory pain, it is still poorly known about the ionic mechanisms that modulate neuronal excitability after inflammation. Sensitization of nociceptive neurons by enhancing the activity of various ion channels can contribute to inflammatory pain (Djouhri et al., 2015; Bennett et al., 2019; Hu et al., 2019; Pitake et al., 2019). Here, we have demonstrated that NALCN enhanced the intrinsic excitability of sensory neurons of the DRG and spinal dorsal horn. Similarly, in other studies, NALCN produced a small background leak Na^+^ current that affected the RMP and regulated neuronal excitability and rhythmic behaviors (Lu et al., 2007; Lu and Feng, 2012; Xie et al., 2013). NALCN increased neuronal excitability and modulated a number of physiological functions, including respiratory rhythms in mice (Lu et al., 2007), locomotion of *C. elegans* (Jospin et al., 2007; Yeh et al., 2008), hypotonia, growth retardation, and the intellectual disability of NALCN mutant children (Cochet-Bissuel et al., 2014; Fukai et al., 2016; Campbell et al., 2018). Ford et al. (2018) reported that NALCN may contribute to neuronal excitability of spinal projection neurons. Additionally, the *C. elegans* NALCN gene, NCA-1, controls the thermal sensitivity of thermo-nociceptor and NCA-2 functions in thermal sensitivity, sensory gain maintenance, and signal kinetics during thermal stimuli termination (Saro et al., 2020). Eigenbrod et al. (2019) showed that highveld mole-rat cells that expressed the NALCN gene produced a depolarized RMP to about −20 mV, which may induce a state unresponsive to normal stimuli. In the present study, relatively small upregulation of NALCN can also lead to depolarization of the RMP by ∼5 mV, which can cause a hyperactive state. Overall, all these studies have provided evidence that NALCN in nociceptors might play a key function in pain processing.

Sodium leak channel and SP are widely expressed and distributed in the DRG and spinal dorsal horn neurons. Lu et al. (2009) reported that SP activated NALCN in the neurons of ventral tegmental area and the hippocampus. Furthermore, SP modulated the stability of the respiratory network and retrotrapezoid nucleus network activity in a NALCN-dependent manner (Yeh et al., 2017). Kim et al. (2012) found that activation of NALCN by SP caused depolarized pacemaking activity in interstitial cells of Cajal. SP is a peptide, produced in peptidergic neurons of the DRG and spinal dorsal horn, which modulates transmission of nociceptive signals (PERNOW, 1953). Spinal dorsal horn neurons also express the NK1 receptor (PERNOW, 1953; Todd, 2002), and the secretion of SP is increased during inflammation. SP increases neuronal excitability through action at NK1 receptors and elicits pain (Navratilova and Porreca, 2019). Like the findings of other studies, we found that SP elicited acute pain-related behaviors after intrathecal injection (Dobry et al., 1981; Share and Rackham, 1981; Partridge et al., 1998; Hua et al., 1999). However, what is the critical target responsible for SP-induced aversive behaviors? Here, we have shown that the pain-related behaviors induced by intrathecal injection of SP were greatly alleviated after the knockdown of the expression of NALCN. Thus, NALCN may be an important molecular target triggering SP-induced aversive behaviors. Our electrophysiology recordings showed that SP-activated NALCN currents were blocked by NK1 receptor antagonist in spinal dorsal horn neurons. These data are in line with the study by Lu et al. in which SP was shown to enhance NALCN conductance via the NK1 receptor (Lu et al., 2009). Our results here show that SP inhibitor and/or NALCN-siRNA produced similar effects for pain relief. Because SP inhibitor was intrathecally injected, therefore, the actions of SP inhibitor or NALCN-siRNA were likely due to a transsynaptic effect of SP from DRG to spinal neurons. Overall, our findings have demonstrated that NALCN is an important background channel for modulating neuronal excitability and is a downstream target for SP-evoked signaling of inflammatory pain. However, further studies are needed to split the changes between NALCN and NK1 receptors and test their specific contribution during inflammatory pain.

Because it is difficult to patch spinal slices of adult animals, we chose to do electrophysiology analysis in the neonate rats. By this design, we can use the rats for both DRG and spinal cord recordings. We also recorded DRG neurons from the adult rats, which is technically available. However, it should be noticed that the physiological function within the sensory circuitry changes with development and the comparison of the recordings from single-isolated DRG neurons at different ages cannot directly apply to the recordings made in the spinal cord. Here, we have proved that NALCN was widely expressed in DRG and spinal cord at both neonatal and adult rats; and NALCN expression was increased in DRG and spinal cord at both ages after CFA injection. All these facts suggests that the comparation between adult and neonatal rats is comparable. Therefore, although electrophysiological recordings in spinal cord slices and *in vivo* therapeutic experiments were not performed in the same rats, the conclusions were deemed reliable.

Small interfering RNA is rapidly effective, but its action duration is short. In our therapeutic experiments, the effects of NALCN-siRNA persisted for 4 days after injection and then the pain behaviors reproduced, most likely because of the degradation of the siRNA. Because the therapeutic efficacy of NALCN-siRNA lasted for ∼3 days, rats at postnatal day 7 were injected with NALCN-siRNA and subsequently injected with CFA3 days later. Then, activity of neurons in the DRG and spinal cord dorsal horn was assayed at 8 and 24 h after CFA injection. Notably, the knockdown of NALCN expression did not change baseline sensory thresholds before injection of CFA, which indicated that baseline NALCN currents in a physiological state might be small and may not have affected normal sensation. Alternatively, the efficacy of the NALCN-siRNA knockdown in the present study may not have been sufficient to change normal sensation. However, NALCN currents were increased after the injection of CFA, and the difference was diminished after treatment of NALCN-siRNA, which indicated that NALCN was probably a target for inflammatory pain.

Western blotting was further used to confirm the change of the NALCN expression level in our study; therefore, the choice of time points for testing the NALCN protein expression was according to the PCR results. In the DRG, the relative mRNA level of NALCN from the CFA-ipsilateral side was significantly increased at 2 h, 8 h, and reached the highest level at 8 h after CFA injection. Therefore, NALCN protein was tested at the 8 h after CFA injection in DRG. For the spinal cord, the relative mRNA level of NALCN from the CFA-ipsilateral side was significantly increased at 2 h, 8 h, 1 day, 3 days, and reached the highest levels at 1 and 3 days after CFA injection; therefore, NALCN protein was evaluated at the 3-day time point after CFA injection in spinal cord.

Our experimental protocols used for recording the NALCN current are according to the previous studies (Yang et al., 2020; Zhang et al., 2021), which are currently the most commonly used. Briefly, extracellular Na^+^ was replaced by NMDG to determine NALCN-mediated background currents, although this cannot completely rule out other Na^+^ currents or other cation currents mediated by NALCN due to our experimental design. Moreover, because of no specific modulators, substances P and Gd^3+^ are widely used as the activator and inhibitor of NALCN conductance, respectively. Nevertheless, we used the siRNA-mediated knockdown of NALCN to further determine the role of NALCN-mediated background currents. In summary, these protocols are typically used to measure NALCN currents.

In this study, we did not use Ca^2+^ channel blockers in patch-clamp recording because the Ca^2+^ channel antagonist is suggested to be a potent blocker of NALCN (Eigenbrod et al., 2019), which may disturb the recording. Besides, Ca_v_ channels were typically activated within the membrane voltage between −40 and +40 mV (Pan et al., 2014; Leo et al., 2017), while, in this study, NALCN currents were mainly recorded at a holding potential of −60 mV. Perhaps, for the same reason, there were also no Ca_v_ blockers used for NALCN currents recordings in previous studies (Lu et al., 2009; Yang et al., 2020; Zhang et al., 2021). However, the low-voltage T-type channels Ca2 + currents, which can be activated from −60 mV, may not be completely ruled out due to our experimental design. For NMDA receptors, it is better to add the NMDA receptor blockers when recording neuronal intrinsic excitability. It is commonly known that NMDAR-mediated excitatory postsynaptic currents were recorded at +40 mV (significant depolarization) in the presence of Mg^2+^ or in the absence of Mg^2+^ (Chen et al., 2016; Ferreira et al., 2017). In our protocol here, the membrane test was recorded at −60 mV with 2 mM Mg^2+^; therefore, it was unlikely to be affected by NMDA receptors even without NMDA blockers in the recording of isolated DRG neurons. However, voltage clamping may not be consistent along with the axon in the spinal cord slices; therefore, only membrane potential cannot fully exclude NMDAR contribution in this network. However, although no Ca^2+^ channel blockers and NMDA receptor blockers were used, we believe that our conclusions make sense by NALCN-siRNA to knockdown the NALCN mRNA. The comparison results between control siRNA and NALCN-siRNA should be contributed by NALCN, although other ion channels and/or receptors can still contribute to both conditions.

In Figure 2C, we aimed to show a morphological profile of NALCN expression in DRG. The results indicated that NALCN was widely expressed in almost all neurons. Therefore, we did not quantify the NALCN fluorescence intensity here because it may be not accurate. Instead, we combined PCR and Western blotting to fully access the expressional level of NALCN. As a result, we did not know the expressional level of NALCN in neuronal subtypes. Also, it may be not necessary to determine the percentage of NALCN positive neurons in different sizes of DRG neurons, because NALCN was present in almost all DRG neurons. Moreover, it is difficult to quantitatively evaluate the difference of the NALCN between different spinal laminae only by immunostaining (e.g., Figure 2D). Future studies that used genetic mice may well determine contribution of NALCN in neuronal subtypes to inflammatory pain.

The present study also has several limitations. Because global NALCN knockout mice cannot survive for behavioral tests, we did not use NALCN knockout mice. Further studies with conditional NALCN knockout mice may better evaluate the function of NALCN in inflammatory pain. In addition, we did not determine which subtype of neurons in the DRG and/or spinal dorsal horn directly accounts for inflammatory pain. Finally, we did not investigate whether NALCN modulates the SP expression in the DRG and/or spinal dorsal horn after inflammatory pain. The underlying interaction between SP expression and NALCN function needs to be further determined.

In summary, this study demonstrated that NALCN is a pivotal ion channel involved in CFA-induced inflammatory pain and neuronal sensitization. Peripheral NALCN may be an effective therapeutic target for inflammatory pain.

## Data Availability Statement

The original contributions presented in the study are included in the article/Supplementary Material, further inquiries can be directed to the corresponding authors.

## Ethics Statement

The animal study was reviewed and approved by the Animal Ethics Committee of West China Hospital of Sichuan University (Chengdu, Sichuan, China) (Approval No. 2018181A).

## Author Contributions

CZ, PL, YZ, and JinL designed the research. JiaL and YC performed the research. DZ, PLL, and JS analyzed the data. JiaL, YC, CM, and CZ wrote the manuscript. All authors read and approved the final manuscript.

## Conflict of Interest

The authors declare that the research was conducted in the absence of any commercial or financial relationships that could be construed as a potential conflict of interest.

## Publisher’s Note

All claims expressed in this article are solely those of the authors and do not necessarily represent those of their affiliated organizations, or those of the publisher, the editors and the reviewers. Any product that may be evaluated in this article, or claim that may be made by its manufacturer, is not guaranteed or endorsed by the publisher.

## Supplementary Material

Supplementary Figure 1 Co-staining of NALCN (red) and SP (green) in spinal dorsal horn after injection of CFA. SP: substance P.

Supplementary Figure 2 The specificity of the NALCN primary antibody was confirmed by Western blotting and fluorescence staining. **(A)** The specificity of the NALCN primary antibody was confirmed by Western blotting. The tissue of spinal dorsal horn was used. Left: spinal dorsal horn was incubated with the NALCN primary antibody. Right: spinal dorsal horn was preincubated with NALCN antigen before the NALCN primary antibody. No specific protein bands were found after preincubation with the NALCN antigen. **(B)** The specificity of the NALCN primary antibody was confirmed by fluorescence staining. The tissue of DRG was used. Left: DRG was incubated with the NALCN primary antibody in the control-siRNA group. Middle: DRG was preincubated with the NALCN antigen before the NALCN primary antibody in the control-siRNA group. No specific staining was observed. Right: DRG was incubated with the NALCN primary antibody in the NALCN-siRNA group. After NALCN-siRNA treatment, the NALCN fluorescence intensity was weaker compared with control-siRNA-treated animals. **(C)** The specificity of the NALCN primary antibody was confirmed by fluorescence staining. The tissue of spinal dorsal horn was used. Left: spinal dorsal horn was incubated with the *n*’s NALCN primary antibody in the control-siRNA group. Middle: spinal dorsal horn was preincubated with the NALCN antigen before the NALCN primary antibody in the control-siRNA group. Right: spinal dorsal horn was incubated with the NALCN primary antibody in the NALCN-siRNA group. After NALCN-siRNA treatment, the NALCN fluorescence intensity was weaker compared with control-siRNA-treated animals. DRG: dorsal root ganglion.

Supplementary Figure 3 Gd^3+^-mediated current inhibition and that produced by siRNA. **(A)** Gd^3+^-mediated current inhibition and that produced by siRNA in adult rats DRG (*n* = 9, by unpaired *t*-test). **(B)** Gd^3+^-mediated current inhibition and that produced by siRNA in PD10 rats DRG (*n* = 9, by Mann–Whitney *U* test). **(C)** Gd^3+^-mediated current inhibition and that produced by siRNA in the spinal cord of PD10 rats (*n* = 9, by unpaired *t*-test). siRNA: NALCN-siRNA. n.s.: no significance.

Supplementary Figure 4 The relative expression of NALCN mRNA in DRG and/or spinal dorsal horn in neonatal rats after injection of CFA (refer to Figures 4–7). **(A)** Similar to adult rats, the level of NALCN mRNA in CFA-ipsilateral side was increased in both DRG (8 h later, *n* = 9, by paired *t*-test) and spinal cord (1 day later, *n* = 9, by paired *t*-test) after injection of CFA in footpad. **(B)** Compared to the rats that received control-siRNA, levels of NALCN mRNA were lower in DRG (*n* = 12, by paired *t*-test) and/or spinal cord (*n* = 11, by Mann–Whitney *U* test) at day 3 after injection of NALCN-siRNA. CFA-contra: CFA-contralateral side; CFA-ipsi: CFA-ipsilateral side; DRG: dorsal root ganglion; data are presented as mean ± SD. ^∗^*p* < 0.05, ^∗∗^*p* < 0.01, ^∗∗∗^*p* < 0.001.

Supplementary Figure 5 Sodium leak channel does not enhance the excitability of large-sized DRG neurons after CFA injection in neonatal rats. **(A,D)** Excitability of large neurons in DRG was compared between CFA-contralateral and CFA-ipsilateral sides based on RMP and rheobase from the rats that received control-siRNA and/or NALCN-siRNA combined with injection of CFA (*n* = 13, Mann–Whitney *U* test). **(B,E)** Action potential in the large neurons of DRG from the rats that received control-siRNA and/or NALCN-siRNA combined with injection of CFA. Red trace is the action potential when receiving the same current injection. **(C,F)** Numbers of APs increased more in small and medium DRG neurons of CFA-ipsi side than those of CFA-contra side from control siRNA-treated rats [**(C)**, *n* = 13, by two-way ANOVA, Holm–Sidak test], but no difference was shown between CFA-ipsi side and CFA-contra side from the NALCN-siRNA-treated rats [**(F)**, *n* = 13, by two-way ANOVA, Holm–Sidak test], respectively. CFA-contra: CFA-contralateral side; CFA-ipsi: CFA-ipsilateral side; DRG: dorsal root ganglion; RMP: rest membrane potential; AP: action potential. Data are presented as mean ± SD or median (first quartile-third quartile). ^∗^*p* < 0.05, ^∗∗^*p* < 0.01, ^∗∗∗^*p* < 0.001, n.s.: no significance.

## Abbreviations

NALCN: sodium leak channel
CFA: complete Freund’s adjuvant
PCR: polymerase chain reaction
DRG: dorsal root ganglion
SP: substance P
NK1: neurokinin 1
TRPV1: transient receptor potential cation channel subfamily V member 1
TTX: tetrodotoxin
PVDF: polyvinyl difluoride
NF200: neurofilament 200
IB4: isolectin B4
Gd^3+^: gadolinium
RMP: resting membrane potential
siRNA: small interfering RNA.
