# Supplementary figures and images for: Elevated Expression and Activity of Sodium Leak Channel Contributes to Neuronal Sensitization of Inflammatory Pain in Rats
Source: Front Mol Neurosci. 2021 Aug 27;14:723395. doi: 10.3389/fnmol.2021.723395 (PMC8430348; doi:10.3389/fnmol.2021.723395)

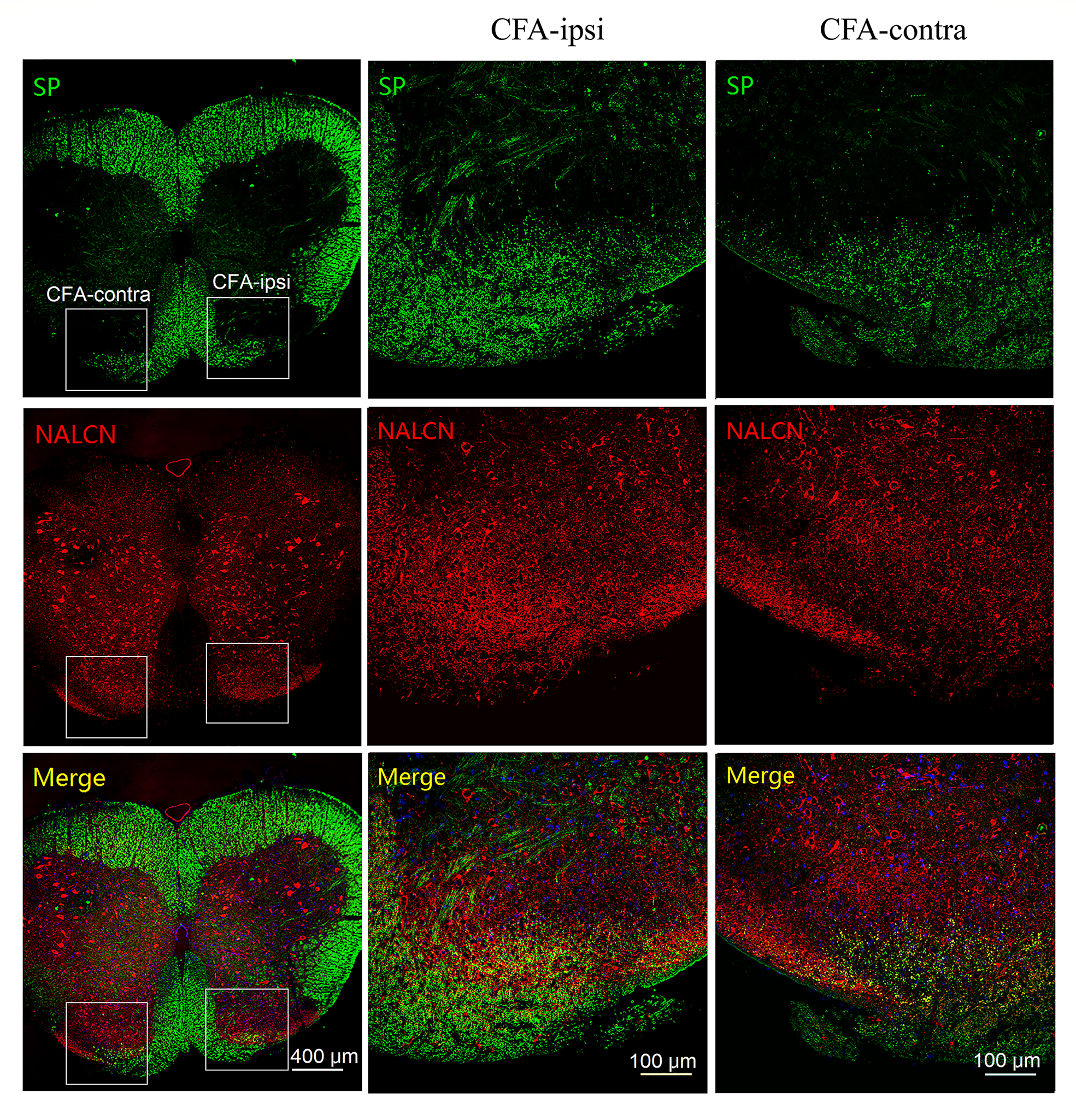

Supplement: Supplementary Figure 1 — Co-staining of NALCN (red) and SP (green) in spinal dorsal horn after injection of CFA. SP: substance P. [file Image_1.TIF]

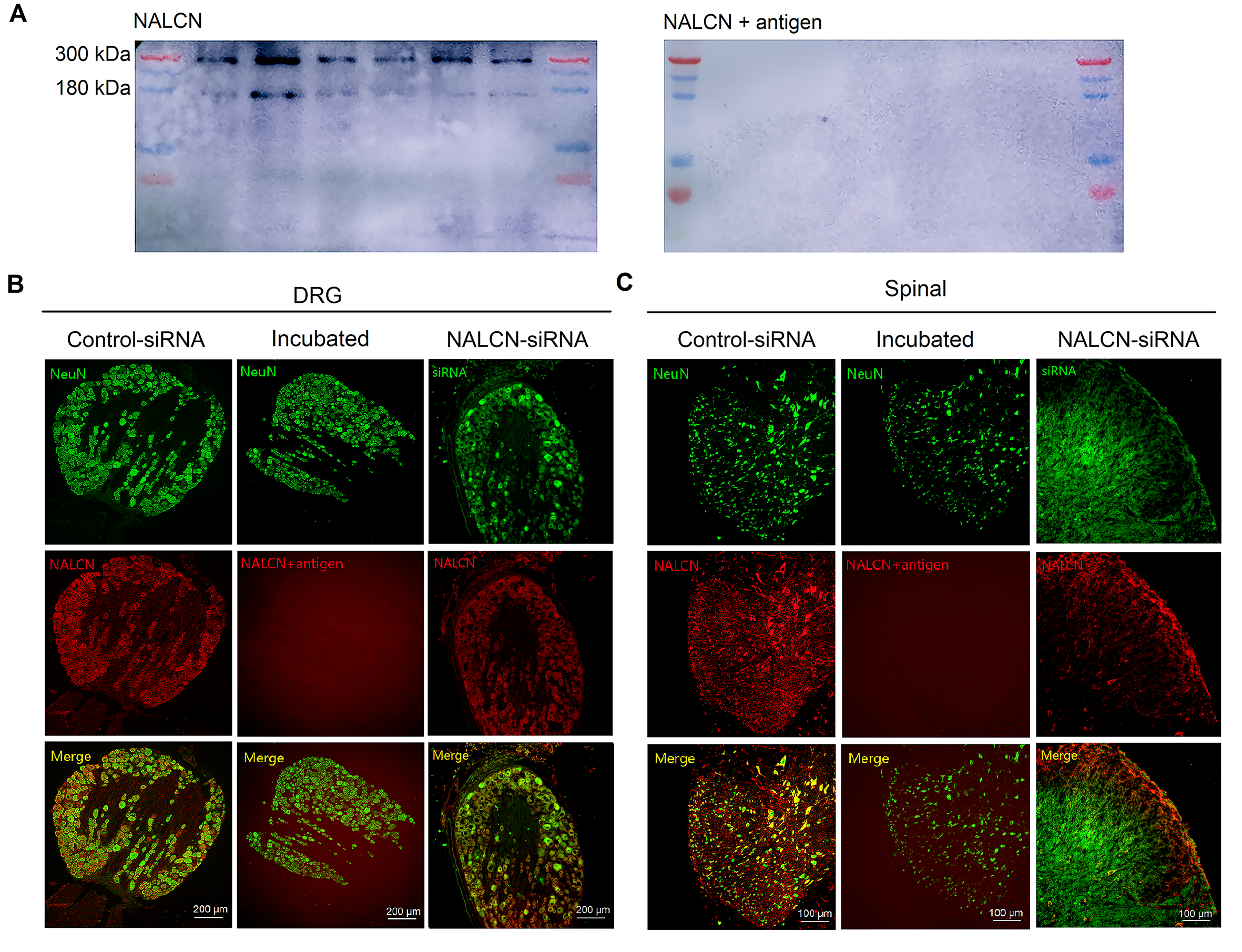

Supplement: Supplementary Figure 2 — The specificity of the NALCN primary antibody was confirmed by Western blotting and fluorescence staining. (A) The specificity of the NALCN primary antibody was confirmed by Western blotting. The tissue of spinal dorsal horn was used. Left: spinal dorsal horn was incubated with the NALCN primary antibody. Right: spinal dorsal horn was preincubated with NALCN antigen before the NALCN primary antibody. No specific protein bands were found after preincubation with the NALCN antigen. (B) The specificity of the NALCN primary antibody was confirmed by fluorescence staining. The tissue of DRG was used. Left: DRG was incubated with the NALCN primary antibody in the control-siRNA group. Middle: DRG was preincubated with the NALCN antigen before the NALCN primary antibody in the control-siRNA group. No specific staining was observed. Right: DRG was incubated with the NALCN primary antibody in the NALCN-siRNA group. After NALCN-siRNA treatment, the NALCN fluorescence intensity was weaker compared with control-siRNA-treated animals. (C) The specificity of the NALCN primary antibody was confirmed by fluorescence staining. The tissue of spinal dorsal horn was used. Left: spinal dorsal horn was incubated with the n’s NALCN primary antibody in the control-siRNA group. Middle: spinal dorsal horn was preincubated with the NALCN antigen before the NALCN primary antibody in the control-siRNA group. Right: spinal dorsal horn was incubated with the NALCN primary antibody in the NALCN-siRNA group. After NALCN-siRNA treatment, the NALCN fluorescence intensity was weaker compared with control-siRNA-treated animals. DRG: dorsal root ganglion. [file Image_2.TIF]

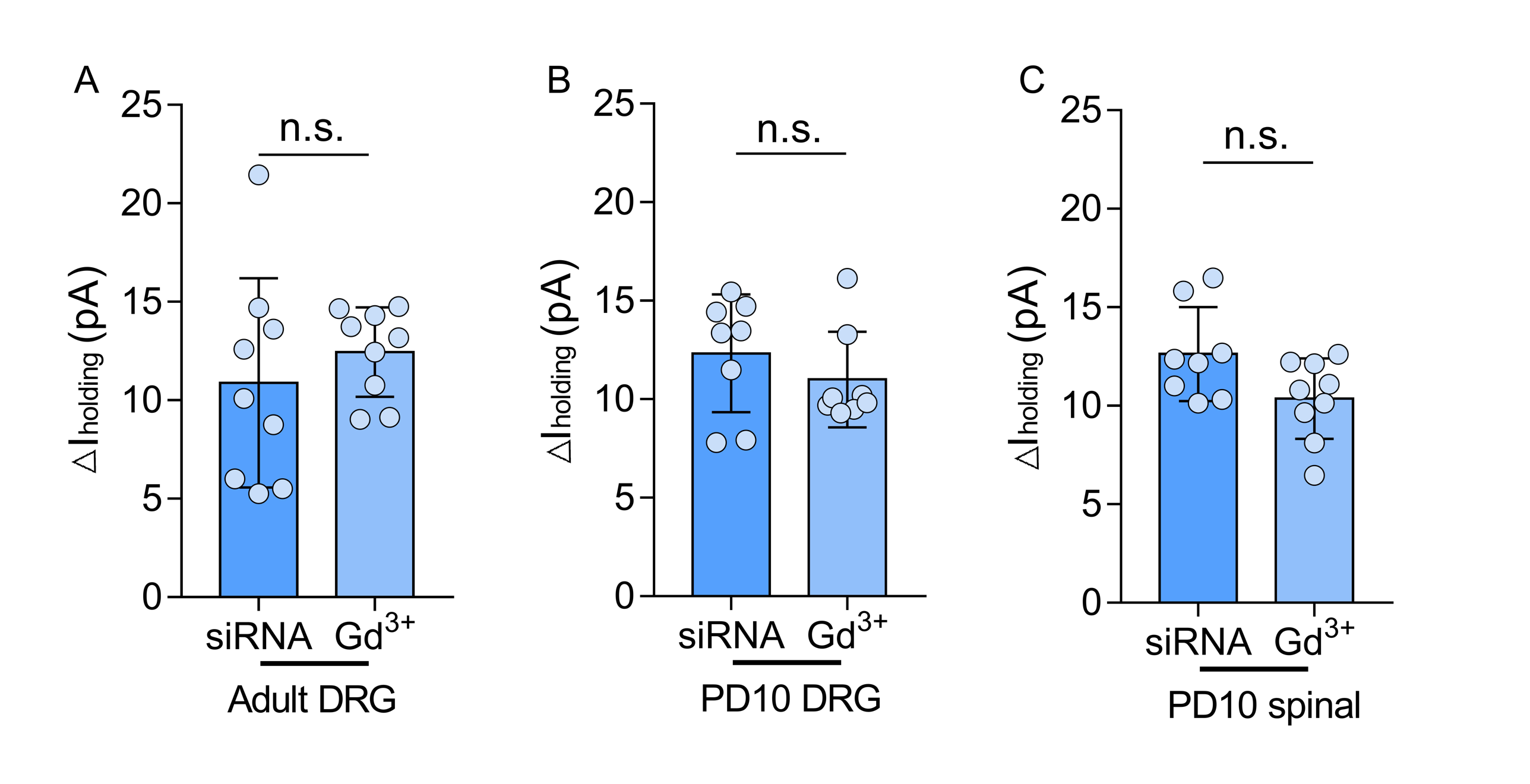

Supplement: Supplementary Figure 3 — Gd3+-mediated current inhibition and that produced by siRNA. (A) Gd3+-mediated current inhibition and that produced by siRNA in adult rats DRG (n = 9, by unpaired t-test). (B) Gd3+-mediated current inhibition and that produced by siRNA in PD10 rats DRG (n = 9, by Mann–Whitney U test). (C) Gd3+-mediated current inhibition and that produced by siRNA in the spinal cord of PD10 rats (n = 9, by unpaired t-test). siRNA: NALCN-siRNA. n.s.: no significance. [file Image_3.JPEG]

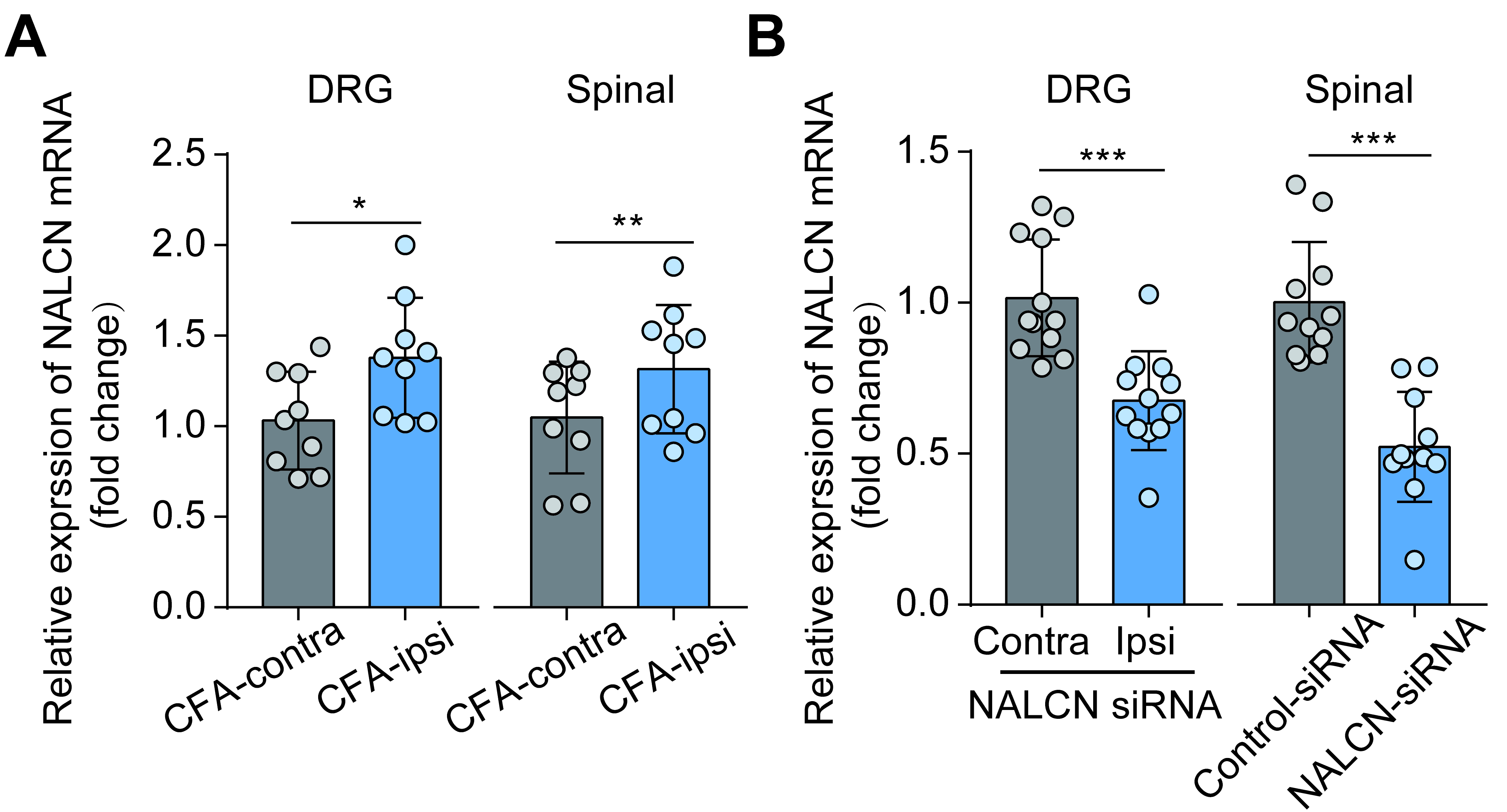

Supplement: Supplementary Figure 4 — The relative expression of NALCN mRNA in DRG and/or spinal dorsal horn in neonatal rats after injection of CFA (refer to Figures 4–7). (A) Similar to adult rats, the level of NALCN mRNA in CFA-ipsilateral side was increased in both DRG (8 h later, n = 9, by paired t-test) and spinal cord (1 day later, n = 9, by paired t-test) after injection of CFA in footpad. (B) Compared to the rats that received control-siRNA, levels of NALCN mRNA were lower in DRG (n = 12, by paired t-test) and/or spinal cord (n = 11, by Mann–Whitney U test) at day 3 after injection of NALCN-siRNA. CFA-contra: CFA-contralateral side; CFA-ipsi: CFA-ipsilateral side; DRG: dorsal root ganglion; data are presented as mean ± SD. ∗p < 0.05, ∗∗p < 0.01, ∗∗∗p < 0.001. [file Image_4.TIF]

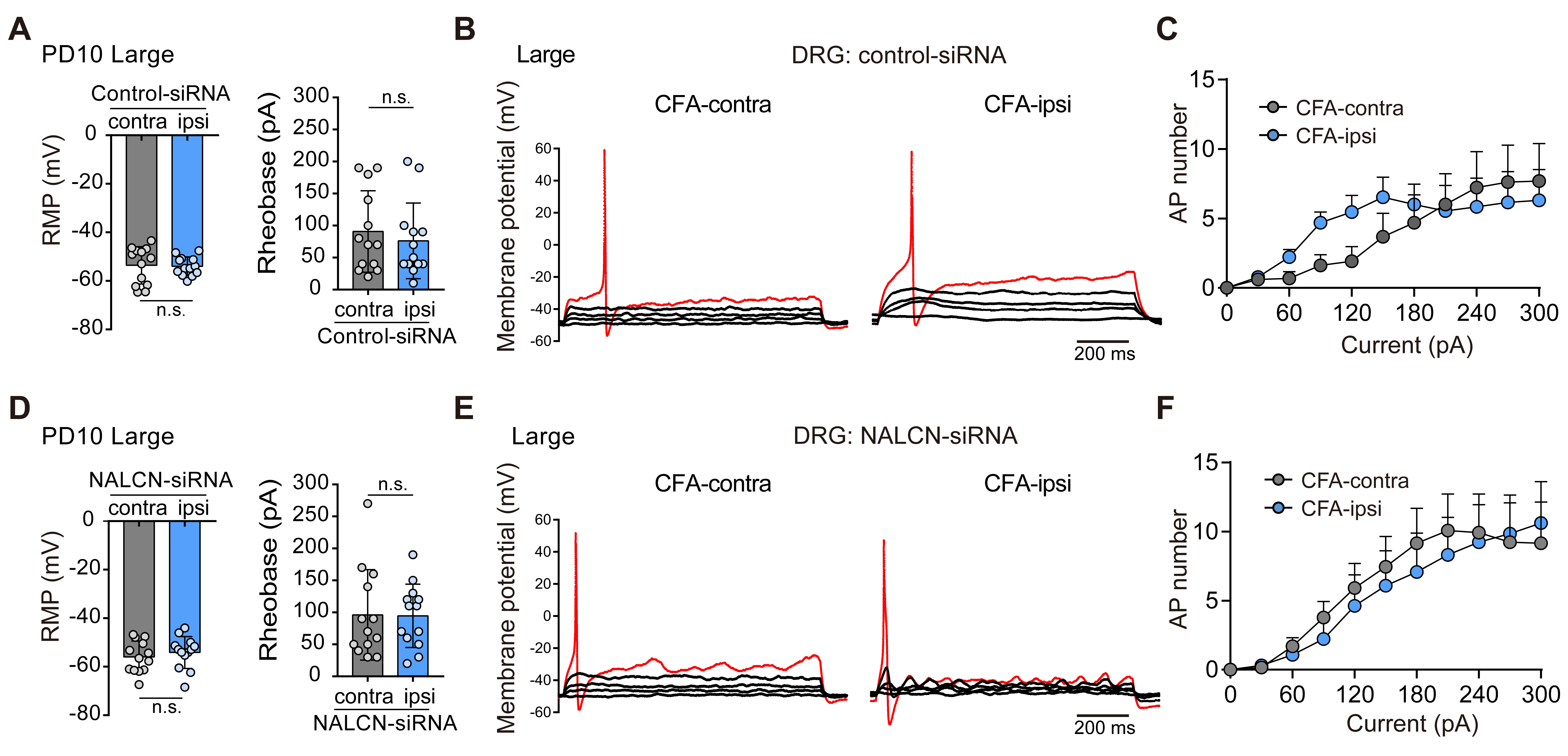

Supplement: Supplementary Figure 5 — Sodium leak channel does not enhance the excitability of large-sized DRG neurons after CFA injection in neonatal rats. (A,D) Excitability of large neurons in DRG was compared between CFA-contralateral and CFA-ipsilateral sides based on RMP and rheobase from the rats that received control-siRNA and/or NALCN-siRNA combined with injection of CFA (n = 13, Mann–Whitney U test). (B,E) Action potential in the large neurons of DRG from the rats that received control-siRNA and/or NALCN-siRNA combined with injection of CFA. Red trace is the action potential when receiving the same current injection. (C,F) Numbers of APs increased more in small and medium DRG neurons of CFA-ipsi side than those of CFA-contra side from control siRNA-treated rats [(C), n = 13, by two-way ANOVA, Holm–Sidak test], but no difference was shown between CFA-ipsi side and CFA-contra side from the NALCN-siRNA-treated rats [(F), n = 13, by two-way ANOVA, Holm–Sidak test], respectively. CFA-contra: CFA-contralateral side; CFA-ipsi: CFA-ipsilateral side; DRG: dorsal root ganglion; RMP: rest membrane potential; AP: action potential. Data are presented as mean ± SD or median (first quartile-third quartile). ∗p < 0.05, ∗∗p < 0.01, ∗∗∗p < 0.001, n.s.: no significance. [file Image_5.TIF]
